# Relationship between default mode network and resting-state electroencephalographic alpha rhythms in cognitively unimpaired seniors and patients with dementia due to Alzheimer’s disease

**DOI:** 10.1093/cercor/bhad300

**Published:** 2023-08-23

**Authors:** Claudio Babiloni, Susanna Lopez, Giuseppe Noce, Raffaele Ferri, Simonetta Panerai, Valentina Catania, Andrea Soricelli, Marco Salvatore, Flavio Nobili, Dario Arnaldi, Francesco Famà, Federico Massa, Carla Buttinelli, Franco Giubilei, Fabrizio Stocchi, Laura Vacca, Moira Marizzoni, Fabrizia D'Antonio, Giuseppe Bruno, Carlo De Lena, Bahar Güntekin, Ebru Yıldırım, Lutfu Hanoğlu, Görsev Yener, Deniz Yerlikaya, John Paul Taylor, Julia Schumacher, Ian McKeith, Laura Bonanni, Patrizia Pantano, Claudia Piervincenzi, Nikolaos Petsas, Giovanni B Frisoni, Claudio Del Percio, Filippo Carducci

**Affiliations:** Department of Physiology and Pharmacology “Vittorio Erspamer,” Sapienza University of Rome, Rome, Italy; Hospital San Raffaele Cassino, Cassino (FR), Italy; Department of Physiology and Pharmacology “Vittorio Erspamer,” Sapienza University of Rome, Rome, Italy; IRCCS Synlab SDN, Naples, Italy; Oasi Research Institute - IRCCS, Troina, Italy; Oasi Research Institute - IRCCS, Troina, Italy; Oasi Research Institute - IRCCS, Troina, Italy; IRCCS Synlab SDN, Naples, Italy; Department of Motor Sciences and Healthiness, University of Naples Parthenope, Naples, Italy; IRCCS Synlab SDN, Naples, Italy; Clinica neurologica, IRCCS Ospedale Policlinico San Martino, Genova, Italy; Dipartimento di Neuroscienze, Oftalmologia, Genetica, Riabilitazione e Scienze Materno-infantili (DiNOGMI), Università di Genova, Italy; Clinica neurologica, IRCCS Ospedale Policlinico San Martino, Genova, Italy; Dipartimento di Neuroscienze, Oftalmologia, Genetica, Riabilitazione e Scienze Materno-infantili (DiNOGMI), Università di Genova, Italy; Clinica neurologica, IRCCS Ospedale Policlinico San Martino, Genova, Italy; Clinica neurologica, IRCCS Ospedale Policlinico San Martino, Genova, Italy; Department of Neuroscience, Mental Health and Sensory Organs, Sapienza University of Rome, Rome, Italy; Department of Neuroscience, Mental Health and Sensory Organs, Sapienza University of Rome, Rome, Italy; IRCCS San Raffaele, Rome, Italy; IRCCS San Raffaele, Rome, Italy; Laboratory of Alzheimer's Neuroimaging and Epidemiology, IRCCS Istituto Centro San Giovanni di Dio Fatebenefratelli, Brescia, Italy; Department of Human Neurosciences, Sapienza University of Rome, Rome, Italy; Department of Human Neurosciences, Sapienza University of Rome, Rome, Italy; Department of Human Neurosciences, Sapienza University of Rome, Rome, Italy; Department of Biophysics, International School of Medicine, Istanbul Medipol University, Istanbul, Turkey; Program of Electroneurophysiology, Vocational School, Istanbul Medipol University, Istanbul, Turkey; Department of Neurology, School of Medicine, Istanbul Medipol University, Istanbul, Turkey; Izmir School of Economics, Faculty of Medicine, Izmir, Turkey; Health Sciences Institute, Department of Neurosciences, Dokuz Eylül University, Izmir, Turkey; Translational and Clinical Research Institute, Faculty of Medical Sciences, Newcastle University, United Kingdom; Translational and Clinical Research Institute, Faculty of Medical Sciences, Newcastle University, United Kingdom; German Center for Neurodegenerative Diseases (DZNE), Rostock, Germany; Translational and Clinical Research Institute, Faculty of Medical Sciences, Newcastle University, United Kingdom; Department of Medicine and Aging Sciences, University “G. d'Annunzio” of Chieti-Pescara, Chieti, Italy; Department of Human Neurosciences, Sapienza University of Rome, Rome, Italy; IRCCS Neuromed, Pozzilli (IS), Italy; Department of Human Neurosciences, Sapienza University of Rome, Rome, Italy; Scuola di Specializzazione in Statistica Medica e Biometria, Dipartimento di Sanità Pubblica e Malattie Infettive, Sapienza University of Rome, Rome, Italy; Laboratory of Alzheimer's Neuroimaging and Epidemiology, IRCCS Istituto Centro San Giovanni di Dio Fatebenefratelli, Brescia, Italy; Memory Clinic and LANVIE - Laboratory of Neuroimaging of Aging, University Hospitals and University of Geneva, Geneva, Switzerland; Department of Physiology and Pharmacology “Vittorio Erspamer,” Sapienza University of Rome, Rome, Italy; Department of Physiology and Pharmacology “Vittorio Erspamer,” Sapienza University of Rome, Rome, Italy

**Keywords:** Structural magnetic resonance imaging (sMRI), Default mode network (DMN), Resting state electroencephalographic (rsEEG) alpha rhythms, Alzheimer’s disease with dementia (ADD), Exact low-resolution brain electromagnetic source tomography (eLORETA)

## Abstract

Here we tested the hypothesis of a relationship between the cortical default mode network (DMN) structural integrity and the resting-state electroencephalographic (rsEEG) rhythms in patients with Alzheimer’s disease with dementia (ADD). Clinical and instrumental datasets in 45 ADD patients and 40 normal elderly (Nold) persons originated from the PDWAVES Consortium (www.pdwaves.eu). Individual rsEEG delta, theta, alpha, and fixed beta and gamma bands were considered. Freeware platforms served to derive (1) the (gray matter) volume of the DMN, dorsal attention (DAN), and sensorimotor (SMN) cortical networks and (2) the rsEEG cortical eLORETA source activities. We found a significant positive association between the DMN gray matter volume, the rsEEG alpha source activity estimated in the posterior DMN nodes (parietal and posterior cingulate cortex), and the global cognitive status in the Nold and ADD participants. Compared with the Nold, the ADD group showed lower DMN gray matter, lower rsEEG alpha source activity in those nodes, and lower global cognitive status. This effect was not observed in the DAN and SMN. These results suggest that the DMN structural integrity and the rsEEG alpha source activities in the DMN posterior hubs may be related and predict the global cognitive status in ADD and Nold persons.

## Introduction

The framework model of the National Institute of Aging (NIA) and Alzheimer’s Association (AA) posit that Alzheimer's disease (AD) diagnosis can be based on biomarkers derived from the in-vivo measurement of amyloidosis (“A”), tauopathy (“T”), and neurodegeneration (“N”) from the brain of patients with AD, regardless of disease clinical manifestations (Jack Jr et al. 2018). Those biomarkers derive from cerebrospinal fluid (CSF) or positron emission tomography (PET) mapping. At the same time, neurodegeneration can be probed by structural magnetic resonance imaging (sMRI) or fluorodeoxyglucose (FDG) PET mapping (Jack Jr et al. 2018).

In the above framework model of AD, there is, unfortunately, no reference to how AD-related neuropathology and neurodegeneration may affect oscillatory neurophysiological thalamocortical mechanisms underpinning abnormalities in the general cortical arousal and vigilance (Hughes and Crunelli 2005; Crunelli et al. 2015), which are common symptoms of AD. Notably, these mechanisms promote the summation of post-synaptic potentials at cortical pyramidal neurons producing detectable changes in the ongoing electromagnetic fields measured at the scalp level during wakefulness (Pfurtscheller and Lopes da Silva 1999). The measurement of these fields unveils the ongoing scalp-recorded electroencephalographic (EEG) rhythms, which have a modest spatial resolution of some squared centimeters but a very high temporal resolution (ms), allowing to investigate cortical EEG rhythms at different frequency bands within about 1–40 Hz during a resting-state eyes-closed condition (rsEEG).

These features are extremely interesting for AD research as biomarkers of the integrity of the neuromodulatory subcortical ascending systems (e.g. reticular formation and noradrenergic, dopaminergic, serotoninergic, and cholinergic pathways) underpinning the disease-related abnormalities in cortical arousal and vigilance regulation (Babiloni et al. 2020, 2021; Rossini et al. 2019). Compared with control cognitively unimpaired old persons (Nold), AD patients with amnestic Mild Cognitive Impairment (ADMCI) and dementia (ADD) were characterized by increased rsEEG rhythms at delta (<4 Hz) and theta (4–7 Hz) frequencies in widespread cortical regions as well as decreased rsEEG rhythms at alpha (8–13 Hz), beta (14–30 Hz), and gamma (30–40 Hz) frequencies distributed in central and posterior cortical areas; these effects were typically discussed concerning the degeneration of cortical synapses and neurons, axonal pathology, and deficits in the cholinergic neurotransmission (Rossini et al. 2019; Babiloni et al. 2020, 2021).

The above rsEEG biomarkers may reflect the effects of AD neuropathology on functional brain networks as revealed by the functional MRI recorded during a resting-state condition in quiet wakefulness (rs-fMRI), based on the measurement of blood oxygenation level-dependent (BOLD) signals; biomarkers of functional connectivity may result from the intrinsic correlation of the BOLD signals recorded between voxels/brain regions (Biswal et al. 1997; Fox and Raichle 2007; Teipel et al. 2016). Among the intrinsic resting-state cortical networks emerging from rs-fMRI, the default mode network (DMN) spanning posterior and anterior cingulate areas, angular gyri, precuneus, and parietotemporal regions is of particular interest in AD research (Heine et al. 2012). Such a network underpins self-related and internal processes that can be parcellated into several sub-classes, including self-awareness or “mental self” and introspection (Gusnard et al. 2001). Specifically, previous rs-fMRI studies showed that AD patients were characterized by decreased DMN activity (Greicius et al. 2004; Zhu et al. 2013; Li et al. 2016) and functional connectivity (Greicius et al. 2004; Zhang et al. 2009; Zhang et al. 2010; Agosta et al. 2012; Koch et al. 2012; Weiler et al. 2014; Xia et al. 2014). Notably, neuroimaging data revealed a fine spatial co-localization of the amyloid-β accumulation and abnormalities in the DMN connectivity in the continuum formed by Nold and AD patients (Myers et al. 2014; Pasquini et al. 2017).

As the generation of rsEEG activity depends on the (de)synchronization of neural activity across brain neural networks (Babiloni et al. 2020), a bulk of rsEEG studies tested the hypothesis of age-related abnormalities in the rsEEG rhythms localized within DMN. Specifically, Caravaglios et al. (2022) showed that compared with Nold participants, amnesic MCI patients were characterized by increased rsEEG delta-beta rhythms (from 21 scalp electrodes) localized in the frontal components of a source DMN model. Exploring the disease progression, Hsiao et al. (2013) found that in contrast with amnesic MCI participants, mild ADD patients showed increased rsEEG delta–theta rhythms and decreased rsEEG alpha–beta rhythms (from 21 scalp electrodes) within DMN source model, with an association between posterior rsEEG theta and alpha rhythms and measures of global cognitive status. Choi et al. (2021) extended those effects with a high spatial-resolution rsEEG approach (from 62 scalp electrodes). Compared with Nold participants, ADD patients were characterized by increased clustering connectivity at the rsEEG theta rhythms and decreased clustering connectivity at the rsEEG alpha rhythms within a source DMN model (Choi et al. 2021). Finally, Brueggen et al. (2017) enriched our understanding of the relationship between the rsEEG rhythms and the DMN in AD with a study using simultaneous recordings of the rsEEG activity (from 19 scalp electrodes) and the rs-fMRI. In contrast to Nold participants, ADD patients showed a reduced correlation between posterior rsEEG alpha rhythms and the activity within the DMN nodes, as revealed by the rs-fMRI (Brueggen et al. 2017).

The above findings indicate a large variability in the relationships between the rsEEG rhythms and the DMN in AD patients. This issue motivated the evaluation of the magnetoencephalographic (rsMEG) counterpart of rsEEG rhythms, which can provide cortical source imaging with a spatial resolution greater than the conventional rsEEG. Unfortunately, those studies confirmed the variability of the relationships of interest. With the rsMEG methodology, Koelewijn et al. (2017) showed that, compared with young and old control persons, mild ADD patients had decreased alpha–beta rhythms (from 275-channel CTF radial gradiometers) in frontoparietal, sensorimotor, and visual cortical networks localized within a source model of the whole brain. Interestingly, those rsMEG effects correlated with global cognitive status measures (Koelewijn et al. 2017).

Similarly, Garcés et al. (2014) reported that, as opposed to Nold persons, MCI patients showed decreased rsMEG alpha rhythms (from 102 magnetometers and 204 planar gradiometers) in a source connectivity DMN model. Furthermore, those MCI patients presented disrupted structural connections among DMN regions, as revealed by the MRI tractography (Garcés et al. 2014). In contrast, Bruña et al. (2022) reported that, compared with Nold persons, MCI patients had increased rsMEG alpha rhythms and decreased rsMEG beta and gamma rhythms (from 102 magnetometers and 204 planar gradiometers) in cingulate and parietal areas of a source connectivity DMN model. This paradoxical increase in the rsMEG alpha rhythms was explained by the relatively younger age of the MCI patients and a very early stage of the disease. Finally, Yu et al. (2017) did not find effects at the rsMEG alpha rhythms when considered separately from the other frequency bands (from a 306-channel whole-head system). ADD patients showed a specific vulnerability in “hubs” localized in the posterior parts of the source DMN model only when the procedure considered all rsMEG frequency bands mixed into a single multiplex network. In that network, such “hubs” vulnerability was associated with altered CSF amyloid-β42 levels and cognitive status (Yu et al. 2017).

Keeping in mind the significant variability in the literature about results on the relationship between the rsEEG rhythms and the DMN in AD patients, we carried out the present exploratory study using a methodological approach based on the correlation between the rsEEG rhythms and the gray matter (GM) volume in the DMN nodes. Specifically, this study was performed in patients with ADD and matched old persons with unimpaired cognitive status (Nold) to test the hypothesis of a specific relationship between (1) the DMN structural integrity, (2) source activities from rsEEG rhythms estimated in the regions of interest (ROIs) representing the DMN core nodes, such as the medial prefrontal cortex-anterior cingulate cortex (mPFC-ACC), the posterior cingulate cortex (PCC), the precuneus, and the inferior parietal cortex (IPC), and (3) the cognitive status, as revealed by the mini-mental state evaluation (MMSE) score.

To evaluate the specificity of the relationship, we used 2 control neural networks such as (1) the dorsal attention network (DAN), which comprises the frontal eye field (FEF) and inferior parietal sulcus (IPS) and may be implicated in directed attention and working memory (Corbetta and Shulman 2002; Fox et al. 2006), and (2) the cortical sensorimotor network (SMN), which groups multiple somatosensory and motor areas, including primary motor cortex (M1, Brodmann area 4, BA 4), caudal premotor (BA 6), and primary somatosensory cortex (S1, BA 3, 1, and 2). This network also includes part of the lateral and medial posterior parietal areas (BA 5 L and BA 5 M) and a small portion of the middle-cingulate sulcus (Yeo et al. 2011). This study’s clinical, MRI, and rsEEG datasets were taken from an international archive, The PDWAVES Consortium (www.pdwaves.eu). The analysis used freeware platforms for MRI and rsEEG data to replicate the study results. Specifically, we used the official eLORETA freeware publicly available at https://www.uzh.ch/keyinst/loreta, which is implemented only with the MNI152 average brain template as a cortical source space (Pascual 2007). This methodological approach will allow any independent research group, including those of lower income countries, to replicate this study, regardless of the availability of individual structural MRIs. Furthermore, this methodological approach allowed us to compare and discuss the present rsEEG results with those obtained in our previous studies using the same approach on patients with mild cognitive impairment due to AD (MCI) and dementia due to other neurodegenerative diseases (e.g. Lewy body and Parkinson’s disease; Babiloni et al. 2020, 2021).

## Materials and methods

### Participants

In this retrospective study of The PDWAVES Consortium (www.pdwaves.eu), clinical and rsEEG data were provided by the following clinical units: Sapienza University of Rome (Italy), IRCCS SDN of Naples (Italy), IRCCS Oasi of Troina (Italy), IRCCS Hospital San Raffaele Pisana of Rome (Italy), University “G. d’Annunzio” of Chieti-Pescara (Italy), Istanbul University (Turkey), Dokuz Eylül University (Turkey), and Newcastle University (UK). Specifically, those data referred to age-, gender-, and education-matched ADD (*n* = 45) and Nold (*n* = 40) participants having rsEEG recordings with consistent eyes-closed conditions. Of note, each clinical unit of this study provided Nold persons and ADD patients. A larger contribution of ADD patients came from the clinical units of Istanbul University, Dokuz Eylül University, and Newcastle University.

The diagnosis of ADD was based on the criteria of the Diagnostic and Statistical Manual of Mental Disorders, fourth edition (DSM-IV-TR; American Psychiatric Association) and the National Institute of Neurological Disorders and Stroke–Alzheimer Disease and Related Disorders (NINCDS–ADRDA; McKhann et al. 2011). Exclusion criteria for the ADD patients were other significant neurological, systemic, or psychiatric illness, mixed dementing diseases, enrolment in a clinical trial with experimental disease-modifying drugs, the chronic use of antidepressant medications, high dose of neuroleptics or frequent user of sedatives or hypnotics, antiparkinsonian medication and the frequent use of narcotic analgesics (Babiloni et al. 2020).

The selected ADD patients underwent the following pharmacological therapies: selective serotonin reuptake inhibitors (SSRIs; *n* = 2; 3.7%), selective serotonin and noradrenaline reuptake inhibitors (SNRIs; *n* = 1; 1.8%), Acetylcholinesterase inhibitors (AChEIs; *n* = 42; 77,8%), and antagonists of N-methyl-D-aspartate receptors (aNMDARs; *n* = 11; 20.4%).

The Nold persons (*n* = 40) were selected from the clinical units in equal percentages of the AD patients to be studied as age-matched controls (19 males, mean age 72.4 years ±1.1 standard error of the mean, SE, a range of 57–87 years). The exclusion criteria for the Nold seniors were (1) the presence of neurological or psychiatric diseases (previous or present), (2) the presence of a condition of depression (detected with a GDS score higher than 5), (3) the use of chronic psychoactive drugs, and (4) significant chronic systemic illnesses (e.g. diabetes mellitus).

All participants received the Mini-Mental State Examination (MMSE) to measure the status of global cognition. Table 1 summarizes the relevant demographic and clinical (i.e. MMSE score) information about the Nold and ADD groups, together with the results of the statistical analyses computed to evaluate the presence or absence of statistically significant differences among them as age (t-test), gender (Fisher’s exact test), education (t-test), and MMSE score (Mann–Whitney U test). As expected, a statistically significant difference was found between the 2 groups for the MMSE score (*P* = 0.000005), showing a higher score in the Nold than in the ADD group. On the contrary, we observed no statistically significant differences in age, gender, and education between the groups (*P* > 0.05).

**Table 1:** Mean values (± standard error, SE) of the demographic and clinical data as well as the results of their statistical comparisons (*P* < 0.05) in the groups of Nold elderly subjects (Nold, *n* = 40) and patients with dementia due to Alzheimer’s disease (ADD, *n* = 45). Legend: M/F = males/females; n.s. = not significant (*P* > 0.05).

	**Nold**	**ADD**	**Statistical comparisons**
**N**	40	45	
**Age (mean ± SE)**	72.4 ± 1.1	74.0 ± 0.9	T-test: n.s.
**Sex (M/F)**	19/21	23/22	Fisher’s exact test: n.s.
**Education (mean ± SE)**	11.0 ± 0.7	10.1 ± 0.5	T-test: n.s.
**MMSE score (mean ± SE)**	27.1 ± 0.2	19.0 ± 0.6	Mann–Whitney U test: *P* < 0.00005

The local institutional Ethical Committees approved the study. All experiments were performed with each participant or caregiver’s informed and overt consent, in line with the Code of Ethics of the World Medical Association (Declaration of Helsinki) and the standards established by the local Institutional Review Board. All experimental data of this study were anonymized in line with the European rules.

### The rsEEG recordings

Electrophysiological data were recorded by professional digital EEG systems licensed for clinical applications. Specifically, the following digital EEG systems were used: BrainAmp 32-Channel DC System (Brain Product GmbH, Germany), Waveguard caps (ANT Neuro, The Netherlands), EB Neuro-BE LIGHT (EB Neuro, Italy), Galileo NT Line—EB Neuro (EB Neuro, Italy), and EB Neuro- Sirius BB (EB Neuro, Italy). The use of different digital EEG systems was properly taken into account in the statistical analysis.

**Fig. 1:**
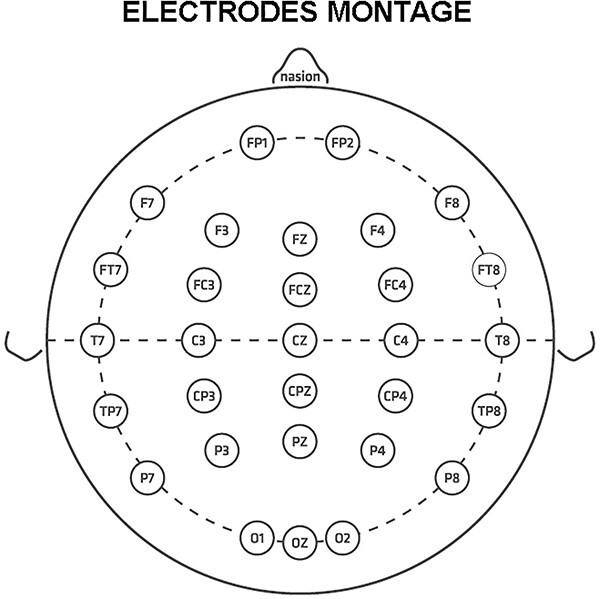
Electrode montage including 30 scalp exploring electrodes placed according to the 10–10 system used in this study.

All rsEEG recordings were performed in the late morning. The rsEEG recordings were performed in all participants using at least 30 scalp exploring electrodes placed according to the 10–10 system. These electrodes were denoted as “selected electrodes,” and their location is illustrated in Fig. 1.

The ground electrode was attached to the right clavicle or on the forehead, while linked earlobes (A1 and A2) or Fz served as the active reference for all the scalp electrodes during the rsEEG recordings. The electrode impedance was kept below 5 kΩ. Continuous EEG data recordings were performed at a 500–1024 Hz sampling frequency and with appropriate antialiasing bandpass filters between 0.01 and 60–100 Hz. The electrooculographic (EOG) potentials (0.3–70 Hz bandpass) were also recorded to control eye movements and blinking.

The participants were seated in a comfortable armchair during the rsEEG recording and instructed to remain awake, psychophysically relaxed (no movement), and with the mind freely wandering (no mental planning or cognitive operations). Based on the instructions given by an experiment, each rsEEG recording lasted 3–5 min in the eyes-closed condition, followed by 3–5 min in the condition of eyes open. The experimenter supervised the participants during the rsEEG recordings to monitor their adherence to the protocol. All deviations by the protocol and verbal interventions between the experimenter and the participants were annotated and considered during the phase of rsEEG data analysis to select artifact-free EEG periods for the source estimation.

### Preliminary rsEEG data analysis

The rsEEG data were centrally analyzed by experts of the Sapienza University of Rome unit; they were blind to the participant’s diagnosis. The recorded rsEEG data were exported as a European data format (.edf) or EEGLAB set (.set) files and then processed offline using the EEGLAB toolbox (Delorme and Makeig 2004; version eeglab14_1_2b) running under the MATLAB software (MathWorks, Natick, MA, USA; version: R2014b). The rsEEG data were divided into epochs lasting 2 s (i.e. 5 min = 150 rsEEG epochs of 2 s each) and analyzed offline.

Afterward, the rsEEG data were analyzed following a 3-step procedure aimed at detecting and removing (1) recording channels (electrodes) showing a prolonged artefactual rsEEG activity due to bad electric contacts or other reasons; (2) rsEEG epochs with artifacts at several recording channels; and (3) intrinsic components of the rsEEG epochs with artifacts.

The first step was based on a visual analysis of the recorded rsEEG activity by 2 independent experimenters among 3 experts (i.e. C.D.P, G.N., and S.L.) for a first identification of the selected electrodes affected by irremediable artifacts. Indeed, no more than 3 selected electrodes were removed for each participant. For the clinical units with a digital EEG system using >30 exploring electrodes, the removed electrodes were substituted with the nearest electrodes not included among the original 30 selected electrodes. The added electrodes were then used together with the artifact-free selected electrodes to compute the interpolation of artifact-free rsEEG data to reconstruct the rsEEG data at the removed electrodes (EEGLAB toolbox, Delorme and Makeig 2004; version eeglab14_1_2b), thus ensuring that all participants had artifact-free EEG data at the locations of the 30 selected electrodes.

The second step was based on a visual analysis of the recorded rsEEG activity by 2 independent experimenters among those involved in the operation (i.e. C.D.P, G.N., and S.L.) to develop a first identification of the artifactual rsEEG epochs. The rsEEG epochs contaminated by muscular, ocular, head movements, or non-physiological artifacts were removed.

The third step was implemented by an independent component analysis (ICA) from the EEGLAB toolbox, applied to remove the ICA components representing the residual artifacts due to the following causes: (1) blinking and eye movements; (2) involuntary head movements; (3) neck and shoulder muscle tensions; and (4) electrocardiographic activity (Jung et al. 2000; Crespo-Garcia et al. 2008). For each rsEEG dataset, less than 5 ICA components were removed from the original ICA solutions based on the working 30 ICA components. In the third step, the rsEEG datasets were reconstructed with the remaining (artifact-free) ICA components, and the putative artifact-free rsEEG epochs were visually double-checked again by 2 independent experimenters (among C.D.P, G.N., and S.L.) to confirm or make the final decision about the inclusion or the exclusion of a given rsEEG epoch.

The artifact-free EEG data for the common 30 selected electrodes were used as input for 2 additional methodological steps. The first additional step served to harmonize rsEEG data recorded by the clinical units using different reference electrodes and sampling frequency rates. The rsEEG data were frequency-band passed at 0.1–45 Hz and downsampled, when appropriate, to make the sampling rate of all artifact-free rsEEG datasets equal 256 Hz. Furthermore, all those rsEEG epochs were re-referenced to the common average reference.

### Spectral analysis of the rsEEG epochs

A standard digital FFT-based analysis (Welch technique, Hanning windowing function, no phase shift) computed the power density of artifact-free rsEEG epochs at all 30 scalp electrodes (0.5 Hz of frequency resolution). From those spectral solutions, the rsEEG frequency bands of interest were individually identified based on the following frequency landmarks: the transition frequency (TF) and individual alpha frequency (IAF) observed in the eyes-closed condition. In the rsEEG power density spectrum, the TF was defined as the minimum rsEEG power density between 3 and 8 Hz, while the IAF was defined as the maximum power density peak between 6 and 14 Hz. These frequency landmarks were previously well described by Dr Wolfgang Klimesch (Klimesch 1996; Klimesch et al. 1998; Klimesch 1999).

The TF and IAF were computed for each participant involved in the study. Based on the TF and IAF, we estimated the individual delta, theta, and alpha bands as follows: delta from TF -4 Hz to TF -2 Hz, theta from TF -2 Hz to TF, low-frequency alpha (alpha 1 and alpha 2) from TF to IAF, and high-frequency alpha (or alpha 3) from IAF to IAF + 2 Hz. Specifically, the individual alpha 1 and alpha 2 bands were computed as follows: alpha 1 from TF to the frequency midpoint of the TF-IAF range and alpha 2 from that midpoint to IAF.

The other rsEEG frequency bands were defined based on the standard fixed frequency ranges used in the reference study series (reviewed in Babiloni et al. 2021): beta 1 from 14 to 20 Hz, beta 2 from 20 to 30 Hz, and gamma from 30 to 40 Hz.

Of note, essential aspects of the procedure were as follows.

(1) Alpha band was divided into sub-bands because, in the rsEEG data, dominant low-frequency alpha rhythms (alpha 1 and alpha 2) may denote the synchronization of diffuse cortical neural networks regulating the fluctuation of the subject’s global wakefulness and vigilance states. In contrast, the high-frequency alpha rhythms (alpha 3) may denote the (de)synchronization of more selective cortical neural networks specialized in processing modal-specific or semantic information during event-related paradigms (Klimesch 1999; Pfurtscheller and Lopes da Silva 1999). When the subject is engaged in sensorimotor or cognitive tasks, alpha and low-frequency beta (beta 1) rhythms do reduce in power (i.e. desynchronization or blocking) and are replaced by fast EEG oscillations at high-frequency beta (beta 2) and gamma rhythms (Pfurtscheller and Lopes da Silva 1999).

(2) We focused on the individual delta, theta, and alpha frequency bands because a mean slowing in the peak frequency of the alpha power density may characterize a clinical group without any substantial change in the magnitude of the power density. In that case, using fixed frequency bands would result in a statistical effect erroneously showing alpha power density values lower in the clinical than in the control group.

(3) Fixed frequency ranges were used for the beta bands because the individual beta frequency peaks were evident only in a few subjects (<10%).

(4) We selected the beginning of the beta frequency range at 14 Hz to avoid overlapping individual alpha and fixed beta frequency ranges (i.e. IAF band ranged from TF to 14 Hz with an IAF = 12 Hz).

### Cortical sources of rsEEG rhythms in the DMN, SMN, and DAN as computed by eLORETA

The procedures for the rsEEG cortical source estimations were described in a previous reference article of our Consortium (Babiloni et al. 2019). We used the official freeware tool called exact LORETA (eLORETA) to linearly estimate the cortical source activity generating scalp-recorded rsEEG rhythms (Pascual 2007). The current implementation of eLORETA uses a head volume conductor model composed of the scalp, skull, and brain.

Exploring electrodes can be virtually positioned in the scalp compartment to give EEG data as an input to the source estimation (Pascual 2007). The brain model relies on a realistic cerebral shape from a template typically used in neuroimaging studies, namely that of the Montreal Neurological Institute (MNI152 template). The eLORETA freeware solves the so-called EEG inverse problem estimating “neural” current density values at any cortical voxel of the mentioned head volume conductor model. The solutions are computed rsEEG frequency bin-by-frequency bin.

The input for this estimation is the EEG spectral power density computed at scalp electrodes. The output estimates the neural current density at the equivalent current dipoles, each localized into one of the 6239 voxels (5 mm resolution) forming the cortical source space, restricted to the cortical GM of the head volume conductor model. Specifically, eLORETA estimates local neural ionic currents at 3 axes, “z,” “x,” and “y,” of a dipolar source located within each voxel of the cortical source space. The procedure averages those values from the 3 axes to make each dipolar source putatively sensitive to different directions of the local neural ionic currents (https://www.uzh.ch/keyinst/loreta). The eLORETA package provides the Talairach coordinates, lobe, and BA for each voxel.

Following the above procedure, the eLORETA source activities from rsEEG rhythms were estimated in specific ROIs representing the main “hubs” included in the resting-state cortical networks considered in this study (i.e. DMN, SMN, and DAN). In this line, the average of the eLORETA source solutions across the voxels of a given ROI could putatively reflect the local neural currents generated by radial, oblique, and tangential rsEEG sources from near cortical circumvolutions, including gyri, sulci, etc. (Pascual 2007).

The selection of the DMN nodes to form the ROIs was performed according to Yeo et al. (2011), while that of the DAN nodes was performed according to Bedini and Baldauf (2021) for the FEFs and to Anderson et al. (2011) for the anterior intraparietal sulcus (aIPS). The correspondence between the network ROIs and the BA is reported in Table 2.

**Table 2:** ROIs used for the estimation of the cortical sources of the rsEEG rhythms in the cortical networks (DMN) explored in this study. Each ROI is defined by some Brodmann areas of the cerebral source space in the freeware used in this study, namely the eLORETA. Legend: mPFC- ACC, PCC, precuneus, IPC, primary somatosensory cortex (S1), primary motor cortex (M1), caudal premotor cortex (PMc), anterior intraparietal sulcus (aIPS).

**BRODMANN AREAS INTO THE NETWORK ROIs**		
**DMN**	**mPFC-ACC**	25, 32, 34
	**PCC**	30, 29, 31, 23
	**Precuneus**	31, 23, 7, 39, 19
	**IPC**	39, 40, 7
**SMN**	**S1**	1, 2, 3
	**M1**	4
	**PMc**	6
**DAN**	**FEF**	part of BA 8 (Bedini and Baldauf 2021)
	**aIPS**	part of BA 7 (Anderson et al. 2011

The following procedure normalized eLORETA solutions computed from the rsEEG eyes-closed data. For a given participant, we averaged the eLORETA solutions across all frequency bins from 0.5 to 45 Hz and 6239 voxels of the brain model volume to obtain the eLORETA “mean” solution. Afterward, we computed the ratio between each original eLORETA solution at a given frequency bin/voxel and the eLORETA “mean” solution. As a result, each original eLORETA solution at a given frequency bin/voxel changed to a normalized eLORETA solution.

For the present eLORETA cortical source estimation, we used a 0.5 Hz frequency resolution as the maximum frequency resolution allowed using 2-s artifact-free EEG epochs.

### MRI data acquisition and anatomical preprocessing

All MRI scans were performed in the Nold and ADD participants using 1.5 and 3.0 Tesla scanners. Each scanner brand was used in the Nold persons and the ADD patients and was considered a bench confound in the group statistical analysis for reproducibility characterization (e.g. General Electric, Philips, Siemens).

The MRI protocol included anatomical T1 scans. The acquired MRI data were anonymized according to international standards to protect sensitive biomedical data. The participant workgroup of the Sapienza University of Rome centrally performed the analysis. Before analyses, all data were visually inspected for quality assurance (i.e. visible artifacts including head motion, wrap-around, radio frequency interference, and signal intensity or contrast inhomogeneities). The MRI data were formatted according to international Brain Imaging Data Structure (BIDS) standards.

Results included in this manuscript come from preprocessing performed using fMRIPrep 20.2.0 (Esteban et al. 2018, 2019), which is based on Nipype 1.5.1 (Gorgolewski et al. 2011). T1-weighted (T1w) images were found within the input BIDS dataset. Each T1w image was corrected for intensity non-uniformity (INU) with N4BiasFieldCorrection (Tustison et al. 2010), distributed with ANTs 2.3.3 (Avants et al. 2008), and used as T1w-reference throughout the workflow. The T1w-reference was then skull-stripped with a Nipype implementation of the antsBrainExtraction.sh workflow (from ANTs), using OASIS30ANTs as the target template. The brain tissue segmentation of CSF, white matter (WM), and GM was performed on the brain-extracted T1w using fast (FSL 5.0.9, Zhang et al. 2001). Brain surfaces were reconstructed using recon-all (FreeSurfer 6.0.1, Dale 1999), and the brain mask estimated previously was refined with a custom variation of the method to reconcile ANTs-derived and FreeSurfer-derived segmentations of the cortical GM of Mindboggle (Klein et al. 2017). Volume-based spatial normalization to one standard space (MNI152NLin2009cAsym) was performed through nonlinear registration with antsRegistration (ANTs 2.3.3), using brain-extracted versions of both T1w reference and the T1w template. The following template was selected for spatial normalization: ICBM 152 Nonlinear Asymmetrical template version 2009c (Fonov et al. 2009; TemplateFlow ID: MNI152NLin2009cAsym). Many internal operations of fMRIPrep use Nilearn 0.6.2 (Abraham et al. 2014), mainly within the functional processing workflow. For more pipeline details, see the workflow section in fMRIPrep’s documentation (https://fmriprep.org/en/20.2.0/workflows.html).

### Cortical network parcellation

Cortical network parcellations were computed following the 7 resting-state cortical networks defined in the Yeo atlas (Yeo et al. 2011). FreeSurfer outputs each network and participant’s average cortical thickness, surface area, and volume. As mentioned above, we focused our analyses on the volume of 3 different cortical networks: the DMN as a main target of the working hypothesis and the DAN and SMN as controls for the specificity of the effects. We also extracted and evaluated the FreeSurfer-derived measures of the total intracranial volume (ICV). This measure was used for each participant to normalize the cortical network volumes, dividing each measurement by their ICV volume.

### Statistical analysis of rsEEG source activities and MRI markers

The statistical analyses of the rsEEG source activities and the MRI markers in the Nold and ADD groups were performed by the STATISTICA software, version 10.0 (StatSoft Inc., www.statsoft.com). Mauchly’s test of sphericity was used to assess whether the assumption of sphericity was met, while the Greenhouse–Geisser correction was applied when the data violated that assumption (Abdi 2015). The Duncan test was used for post-hoc comparisons (*P* < 0.05, Bonferroni corrected).

As the use of analysis of variance (ANOVA) models implies that the dependent variable must be normally distributed, the Kolmogorov–Smirnov test (*P* < 0.05) was used to determine if the regional normalized eLORETA rsEEG current density distributions (i.e. the eLORETA source activities) of a given ANOVA model approximated to Gaussian distributions (null hypothesis of non-Gaussian distributions tested at *P* < 0.05). This prerequisite was not true in some cases, so all regional eLORETA source activities were used as inputs to the log 10 transformation to make the eLORETA solutions Gaussian. The Kolmogorov–Smirnov test confirmed that all eLORETA regional solutions were Gaussian after that transformation (*P* > 0.05).

The MRI variables respected the assumption of normality data distribution according to the Kolmogorov–Smirnov test (*P* > 0.05).

Three statistical sessions were performed. The first statistical session tested the working hypothesis that the MRI markers of normalized GM volume in the DMN, DAN, and SMN may differ between the Nold and ADD groups. An ANOVA was computed using the normalized GM volume as a dependent variable to address this hypothesis. That ANOVA used the following factors: Group (Nold and ADD) and Network (DMN, DAN, SMN). The Clinical Unit (recording site) was used as a covariate. The confirmation of the working hypothesis may require (i) a statistically significant ANOVA effect including the factor Group (*P* < 0.05) and (ii) a post-hoc Duncan test indicating statistically significant (*P* < 0.05, Bonferroni corrected) differences in the normalized GM volume between the Nold and ADD groups (i.e. Nold ≠ ADD).

The second session tested the hypothesis that the rsEEG source activities may differ between the Nold and ADD groups among the different ROIs of each resting-state cortical network of interest. To this aim, for each cortical network (DMN, SMN, DAN), one ANOVA was computed using the normalized eLORETA solutions in the specific network ROIs as a dependent variable. The ANOVA factors were Group (Nold, ADD), network-ROIs (as previously defined), and Band (delta, theta, alpha 2, alpha 3, beta 1, beta 2, and gamma). The Clinical Unit (recording site) was used as a covariate. The confirmation of the working hypothesis may require (1) a statistically significant ANOVA interaction including the factor Group (*P* < 0.05) and (2) a post-hoc Duncan test indicating statistically significant (*P* < 0.05, Bonferroni corrected) differences in the rsEEG source activities estimated from network-ROIs between the Nold and ADD groups at the delta and alpha bands, typically affected in the ADD patients using eLORETA rsEEG source estimation and large lobar ROIs (Babiloni et al. 2020, 2021).

The third session used several linear regression models. They evaluated the interacting effect between rsEEG source activity from each specific network-ROI and the corresponding GM network volume to predict the global cognitive status (MMSE score) in the Nold and ADD participants. More specifically, they tested the 2-way interacting effect of the following predictors on the MMSE score, considering the ADD and Nold participants as a whole group:

- The rsEEG cortical source activity in each network-ROI (only those showing statistically significant post-hoc differences between the ADD and Nold groups in the previous analysis, *P* < 0.05);
- The corresponding GM network volume.

## Results

### Cortical networks parcellation

**Fig. 2:**
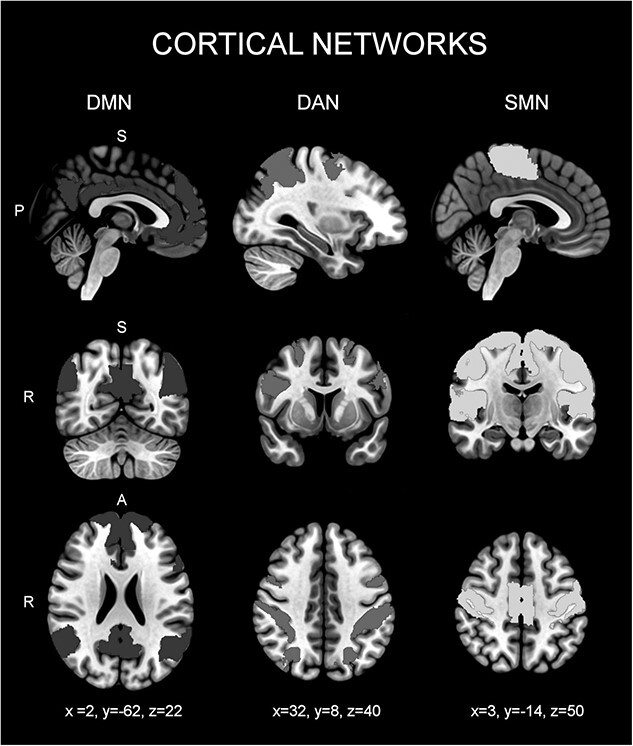
Sagittal, coronal, and axial images showing the spatial distribution of the DMN, DAN, and SMN, superimposed on a standard MNI152 T1 brain template. The network map distributions are based on a clustering approach to identify and replicate networks of functionally coupled regions across the cerebral cortex.

Figure 2 shows the spatial distribution of the 3 resting-state cortical networks, taken from Yeo’s atlas, used in the current study. The DMN included the PCC, precuneus (PCUN), mPFC, and IPC (Buckner et al. 2008; Andrews-Hanna et al. 2010; Raichle 2015). The DAN comprised the FEF and IPS (Corbetta and Shulman 2002; Fox et al. 2006). The SMN grouped multiple somatosensory and motor areas, including the primary motor cortex (M1, BA 4), caudal premotor (BA 6), and primary somatosensory cortex (S1, BA 3, 1, and 2; Yeo et al. 2011). This network also included most (if not all) of the early somatosensory area BA 5 L, a small portion of the midcingulate sulcus, and possibly part of BA 5 M (Yeo et al. 2011).

### MRI markers of the normalized network GM volume in Nold and ADD groups

**Fig. 3:**
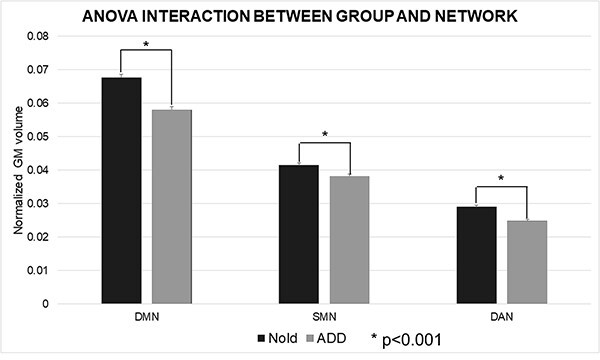
DMN, SMN, and DAN normalized GM volumes (mean across subjects ± standard error, SE) relative to a statistical ANOVA interaction (F(2, 164) = 31.524, *P* < 0.001; unit as a covariate) among the factors group (Nold elderly subjects, Nold, *n* = 40; Alzheimer’s disease patients with dementia, ADD, *n* = 45) and these cortical networks. Individual ICV was used to normalize network volumes for head size.

The results of the first statistical session about the MRI markers in all Nold and ADD participants are illustrated in Fig. 3. The ANOVA evaluating the differences in the GM volume of the cortical networks of interest between the Nold and ADD groups showed a statistically significant interaction effect (F (2, 166) = 31.5; *P* < 0.001; Clinical Unit as a covariate) among the factors Group (Nold and ADD) and Network (DMN, SMN, and DAN). The Duncan planned post-hoc testing (*P* < 0.05 Bonferroni correction for 2 groups X 3 Networks, *P* < 0.05/6 = 0.00833) revealed that the discriminant pattern Nold > ADD was fitted by all the 3 cortical networks considered DMN (*P* < 0.0001), SMN (*P* < 0.001), and DAN (*P* < 0.001) networks but with more marked mean differences with the DMN.

### Individual frequencies and distribution of posterior rsEEG source activities in the Nold and ADD groups

The mean TF was 5.7 Hz (± 0.1 SE) in the Nold group (*n* = 40) and 5.5 Hz (± 0.2 SE) in the ADD group (*n* = 45). Furthermore, the mean IAF was 8.9 Hz (± 0.1 SE) in the Nold group and 8.4 Hz (± 0.2 SE) in the ADD group. The T-tests of these data showed that the mean IAF was greater in the Nold than in the ADD groups (*P* < 0.05). No statistically significant difference was found for the mean TF (*P* > 0.05).

**Fig. 4:**
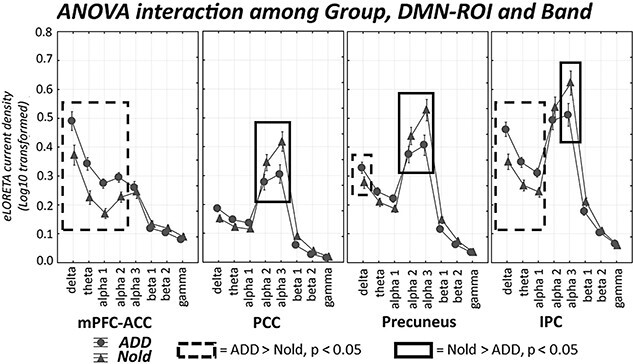
DMN-ROI rsEEG source activity (mean across subjects ± standard error, SE) relative to a statistical ANOVA interaction (F (21, 1722) = 4.16, *P* < 0.0001; clinical unit as a covariate) among the factors group (Nold elderly subjects, Nold, *n* = 40; Alzheimer’s disease patients with dementia, ADD, *n* = 45), DMN-ROIs (mPFC-ACC, PCC, Precuneus, IPC), and bands (delta, theta, alpha 1, alpha 2, alpha 3, beta 1, beta 2, and gamma. This ANOVA design used the regional rsEEG eyes-closed normalized eLORETA solutions as a dependent variable. The rectangles indicate the cortical regions and frequency bands in which the posterior eLORETA solutions (rsEEG source activities) presented a statistically significant pattern Nold ≠ ADD (*P* < 0.05 uncorrected).

**Fig. 5:**
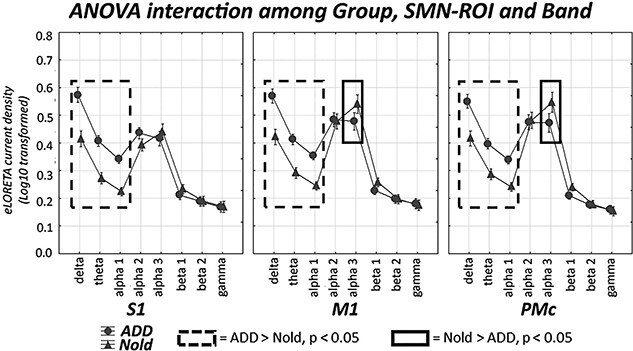
SMN-ROI rsEEG source activity (mean across subjects ± standard error, SE) relative to a statistical ANOVA interaction (F(14, 1148) = 2.0566, *P* < 0.05; unit as a covariate) among the factors group (Nold elderly subjects, Nold, *n* = 40; Alzheimer’s disease patients with dementia, ADD, *n* = 45), SMN-ROIs (S1, M1, PMc), and bands (delta, theta, alpha 1, alpha 2, alpha 3, beta 1, beta 2, and gamma). This ANOVA design used the regional rsEEG eyes-closed normalized eLORETA solutions as a dependent variable. The rectangles indicate the cortical regions and frequency bands in which the posterior eLORETA solutions (rsEEG source activities) presented a statistically significant pattern Nold ≠ ADD (*P* < 0.05 uncorrected).

**Fig. 6:**
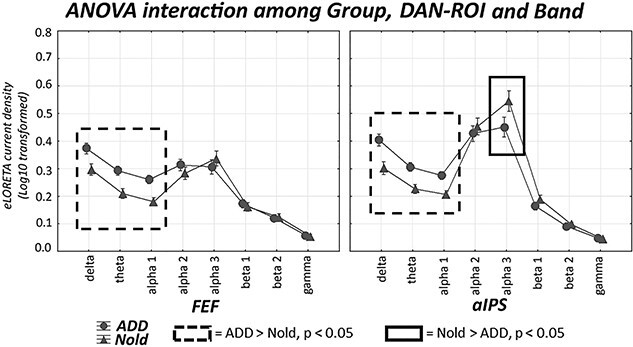
DAN-ROI rsEEG source activity (mean across subjects ± standard error, SE) relative to a statistical ANOVA interaction (F(7, 574) = 3.6085, *P* < 0.001; unit as a covariate) among the factors group (Nold elderly subjects, Nold, *n* = 40; Alzheimer’s disease patients with dementia, ADD, *n* = 45), DAN-ROIs (FEF, aIPS), and bands (delta, theta, alpha 1, alpha 2, alpha 3, beta 1, beta 2, and gamma). This ANOVA design used the regional rsEEG eyes-closed normalized eLORETA solutions as a dependent variable. The rectangles indicate the cortical regions and frequency bands in which the posterior eLORETA solutions (rsEEG source activities) presented a statistically significant pattern Nold ≠ ADD (*P* < 0.05 uncorrected).

The results of the second statistical session concerned the rsEEG source activities estimated in the network ROIs in all Nold and ADD participants and are illustrated in Figs. 4, 5, and 6. These figures show the mean values (± SE, Log10 transformed) of the rsEEG source activities estimated in the network-ROIs (as revealed by normalized eLORETA solutions) for the comparison between the Nold (*n* = 40) and ADD (*n* = 45) groups. The results showed that the distribution of those rsEEG source activities differed among the groups, network ROIs, and frequency bands. In the Nold group, as a physiological reference, the (eLORETA) rsEEG alpha 2 and 3 source activities showed dominant values over the other frequency bands in several posterior network-ROIs: in the PCC, precuneus, and IPC for the DMN (Fig. 4); in the M1 and PMc for the SMN (Fig. 5); and in the aIPS for the DAN (Fig. 6). In the same network ROIs, the rsEEG delta and theta source activities were characterized by relatively low values, while the rsEEG beta1, beta 2, and gamma source activities were generally very low in magnitude.

The ANOVA results showed a statistical interaction effect for the 3 resting-state cortical networks of interest (DMN: F(21, 1722) = 4.157, *P* < 0.0001; SMN: F(14, 1148) = 2.057, *P* < 0.05; DAN: F(7, 574) = 3.608, *P* < 0.001) among the factors Group (Nold and ADD), network-ROI (as defined above), and Band (delta, theta, alpha 1, alpha 2, alpha 3, beta 1, beta 2, and gamma). The Duncan planned post-hoc (*P* < 0.05 uncorrected) testing showed that compared with the Nold group, the ADD group exhibited the following effects:

- Increased rsEEG source activities from delta to alpha 2 bands in the mPFC-ACC, along with increased delta rsEEG source activities in the precuneus and rsEEG source activities from delta to alpha1 bands in the IPC (ADD > Nold, *P* < 0.005);
- Decreased rsEEG alpha 2 and alpha 3 source activities in the PCC, Precuneus, and IPC DMN-ROIs (ADD < Nold, *P* < 0.00005 to *P* < 0.05);
- Increased rsEEG delta, theta, and alpha1 source activities in all SMN and DAN-ROIs (ADD > Nold, *P* < 0.00005);
- Decreased rsEEG alpha 3 source activities in the M1 and PMc SMN-ROIs and the aIPS DAN-ROI (ADD < Nold, *P* < 0.005).

These findings were not due to outliers from those individual regional normalized eLORETA current densities (log 10 transformed), as shown by the results of the Grubbs’ test set with an arbitrary threshold of *P* > 0.001.

For the third statistical session, we included the rsEEG source activities within network ROIs showing statistically significant differences between the Nold and ADD groups as predictors in the linear regression models (*P* < 0.05). More specifically, the following rsEEG source activities were included: the PCC, Precuneus, and IPC for rsEEG alpha 2 and alpha 3 source activities; the mPFC-ACC, S1, M1, PMc, FEF, and aIPS for the rsEEG delta–theta-alpha 1 (average of the 3 frequency bands) source activities; the mPFC-ACC for the rsEEG alpha 2 source activity; the M1, PMc, and aIPS for the rsEEG alpha 3 source activity. The results of this analysis are illustrated in Table 3. Only for the DMN, statistically significant 2-way interaction effects were observed (*P* < 0.05).

**Table 3:** Statistically significant results of linear regression models showing the effect on the global cognitive status (MMSE) dependent variable of 2-way interaction between the predictors DMN-ROI rsEEG source activity and the DMN GM volume. The selected network-ROI rsEEG source activities showed statistically significant differences between the Nold and ADD groups in the previous ANOVAs (Duncan’s post-hoc, *P* < 0.05). Only statistically significant 2-way interaction effects are reported (*P* < 0.05). Only for DMN, statistically significant 2-way interaction effects were observed to predict the MMSE score. No statistically significant interacting effect between SMN-ROI and DAN-ROI rsEEG source activity and GM network volume. Legend: GMVn = normalized gray matter volume.

	**F value**	***P*-value**	**β coefficient**	**SE**
***Alpha2 PCC*** *DMN_GMVn	6.2213	0.01466	230.564	92.438
***Alpha3 PCC*** *DMN_GMVn	9.4478	0.002880	239.672	77.974
***Alpha2 Precuneus*** *DMN_GMVn	4.8430	0.03061	204.64	92.99
***Alpha3 Precuneus*** *DMN_GMVn	7.1090	0.009257	197.727	74.159
***Alpha3 IPC*** *DMN_GMVn	5.9128	0.01724	171.777	70.643

In the Supplementary Materials, we reported the results of the control analyses exploring the following effects:

- Distribution of the posterior rsEEG source activity estimated in the Nold and ADD groups (Fig. SM1);
- Correlation analysis between the MRI markers of the resting-state cortical ROIs and the posterior rsEEG source activity (Fig. SM2 and Table SM2);

Furthermore, Figs. SM3, 4, and 5 report the scatterplots of the above results, while Table SM3 reports all coefficients and *P*-values relative to the correlation analyses showing significant associations between pairs of the following variables measured in the Nold persons and ADD patients considered as a whole group:

- Global cognition (MMSE, score)
- Neurodegeneration in the DMN, DAN, and SMN GM volumes
- rsEEG posterior source activities.

## Discussion

### A new approach investigating the relationship between DMN structural integrity and the cortical rsEEG rhythms

Here we used a new methodological approach to explore the relationship between the DMN structural integrity, cortical rsEEG rhythms recorded in the resting-state condition, and the cognitive status in Nold and ADD participants. The new approach was based on the correlation analysis between the structural MRI biomarkers measuring the DMN GM volume, the regional (lobar) rsEEG source activities estimated from 30 scalp electrodes within the DMN nodes, and the MMSE score. In this framework, the DAN and SMN served as control resting-state cortical networks to test the specificity of that relationship. To promote the results’ replicability, WEB-based freeware procedures were used to extract structural MRI and rsEEG source markers (see Methods for details). This new multimodal structural MRI-rsEEG approach may partially overcome the intrinsic limitation in spatial resolution of rsEEG techniques, partially mitigating uncertainties in the discrimination of rsEEG sources located in some small cortical components of the DMN.

The results of this study showed that, compared with the Nold group, the ADD group was characterized by a significant positive association between the GM volume in the DMN, the rsEEG alpha source activity estimated in the posterior DMN nodes (parietal and PCC), and the global cognitive status (MMSE score) in the Nold and ADD participants. Compared with the Nold group, the ADD group showed lower DMN GM, lower rsEEG alpha source activity in those nodes, and lower global cognitive status. This effect was not observed in the DAN and SMN.

The results of the present structural MRI-rsEEG approach emphasize the strict relationship between neurodegeneration in the posterior DMN nodes and related abnormalities in the rsEEG alpha rhythms in AD patients. They complement previous findings derived from estimating rsEEG source activities into mathematical models of the DMN in AD patients (using rsEEG activity recorded from 19 to 62 scalp electrodes as an input for a cortical rsEEG source imaging). Specifically, they agree with previous findings showing that in comparison with Nold persons, MCI and ADD patients were characterized by increased rsEEG delta–theta source activities located in DMN regions of the source models (Hsiao et al. 2013; Sheorajpanday et al. 2013; Choi et al. 2021; Caravaglios et al. 2022). In contrast, the present results disagree with findings pointing to reduced rsEEG alpha–beta source activities in those DMN regions (Hsiao et al. 2013; Choi et al. 2021; Caravaglios et al. 2022).

Similarly, the present results complement previous findings on estimating rsMEG source activities into mathematical models of the DMN in AD patients (using rsMEG activity recorded from >100 extracranial recording sensors as an input for a cortical rsEEG source imaging). Specifically, they agree with previous findings showing that as opposed to Nold persons, MCI and ADD patients were characterized by no relationship between rsMEG alpha rhythms and DMN regions of source models when those rhythms were considered separately from the other frequency bands (Yu et al. 2017). In this line, other rsMEG findings showed that the association with alpha–beta rhythms regarded other cortical networks, including frontoparietal, sensorimotor, and visual cortical regions, within a source model of the whole brain (Koelewijn et al. 2017). Furthermore, the association between the rsMEG alpha rhythms and the DMN in AD patients may rely on the source connectivity rather than the mere source activity as reported in other studies (Bruña et al. 2022), even in correlation with disrupted structural connections among DMN regions (Garcés et al. 2014). Indeed, there was large heterogeneity in the procedures previously used for the rsEEG/MEG source estimation, the analysis of rsEEG/MEG source connectivity within the DMN, and the fragmentation in ROIs within the DMN. Moreover, another source of variability in those previous studies was the enrollment of Nold, MCI, and ADD patients with significant age, sex, and education differences.

### The neurophysiological model on the generation of rsEEG delta rhythms in the DMN

The present results show a positive association between the DMN structural integrity and the related posterior rsEEG alpha source activities in the Nold and ADD participants, which could reflect the typical “slowing” of the rsEEG rhythms related to the age-related neurodegenerative cortical process (i.e. synaptic and neuronal loss), accelerated by the AD course (Smailovic and Jelic 2019; Babiloni et al. 2021). At the early stage of this research line, we can just provide the following speculative explanation of the present results as a seed for future explorative studies.

The relationship between rsEEG alpha rhythms and brain neural circuits has been debated. It was proposed that in physiological conditions, scalp rsEEG alpha rhythms may reflect the oscillatory neural activity along cortico-thalamic and thalamocortical loops that functions in inhibiting cortical information processing across local sensory, motor, and posterior associative areas (e.g. selective attention/intentions and expectancy) as well as wide cortical neural networks inducing quiet levels of vigilance/consciousness with mind wandering, introspection, etc. (Nunez et al. 1994; Klimesch 1999; Pfurtscheller and Lopes da Silva 1999; Babiloni et al. 2003).

In pathophysiological conditions characterizing several brain diseases (including AD and other age-related neurodegenerative diseases), posterior rsEEG alpha rhythms have low amplitude and are associated with abnormalities in DMN and cognitive functions (Hsiao et al. 2013; Brueggen et al. 2017; Choi et al. 2021). Furthermore, decreased rsEEG alpha rhythms were related to the following molecular, neuroanatomical, and pathophysiological changes in the AD patients’ brains: (1) the Cystatin C genotyping as an independent genetic risk of sporadic AD (Babiloni et al. 2006a); (2) the level of the neurotoxic free copper in the blood (Babiloni et al. 2007); (3) vascular lesions in the subcortical WM measured by MRIs (Babiloni et al. 2006b); and (4) the normalized GM volume measured in the cerebral cortex by volumetric MRIs (Babiloni et al. 2013).

Animal studies elucidated the neurophysiological basis of how AD neuropathology may affect the functioning of cortical neural networks increasing the generation of slow-frequency EEG rhythms. It was reported that in transgenic mice, an early abnormal circulation of amyloid-b protein in the brain (before the formation of amyloid plaques) perturbed cortical neural networks, as revealed by fMRI measures, while an anti-amyloid treatment prevented behavioral abnormalities (Shah et al. 2016). It was also reported that transgenic mice producing an abnormal amyloid accumulation in the brain showed altered EEG delta rhythms recorded in wakefulness (Del Percio et al. 2018, 2020), which were modulated by cholinergic but not anti-amyloid drugs (Lopez et al. 2020). Concerning the other AD neuropathological hallmark, it was found that mice receiving the inoculation of human tau protein in the brain exhibited abnormal slow-frequency EEG rhythms recorded in memory-related brain networks (Ahnaou et al. 2017).

Other hints on the relationship between AD neuropathology, cortical networks, and rsEEG delta rhythms come from the European “The Virtual Brain” project (https://www.thevirtualbrain.org/tvb/zwei; Ritter et al. 2013). This project developed a virtual human brain model incorporating a priori neurobiological, neuroanatomical, and neurophysiological knowledge enabling it to predict the effects of AD-related amyloid plaques on the excitatory/inhibitory balance in the cortical pyramidal cells and interneurons that result in the “slowing” in rsEEG rhythms like those quite often found in ADD patients (Stefanovski et al. 2019).

Overall, these data and considerations suggest that AD-related neuropathology might induce pathophysiological abnormalities in excitatory/inhibitory balance, neural signaling, and synaptic neurotransmission in cortical and subcortical networks with high intrinsic connectivity, including the DMN and ascending arousing cholinergic systems. These pathophysiological abnormalities may be responsible for a functional disconnection within those neural networks reflected by exaggerated rsEEG delta rhythms and reduced rsEEG alpha rhythms in quiet wakefulness (Myers et al. 2014; Shah et al. 2016;Teipel et al. 2016; Ahnaou et al. 2017; Pasquini et al. 2017; Del Percio et al. 2018, 2020; Lopez et al. 2020; Babiloni et al. 2021).

Finally, the present results unveiled the strict relationship in AD between the well-documented functional cortical dysconnectivity and underlying neurophysiological mechanisms oscillating at alpha frequency. Indeed, a bulk of structural, molecular, and functional neuroimaging studies (Bozzali et al. 2001, 2002; Chételat et al. 2002, Chételat et al. 2013; Choo et al. 2007; Whitwell et al. 2007; Thal et al. 2014; van den Heuvel et al. 2009; Zhu et al. 2013; Babiloni et al. 2020, 2021) previously reported that compared with Nold seniors, patients with MCI and mild dementia due to AD showed poor (1) callosal, thalamic, and anterior–posterior WM bundles; (2) cortical GM volume in association with in-vivo neuroimaging maps of abnormal deposition of tau and amyloid; (3) correlation of resting-state functional MRI-BOLD signal in the DMN and DAN; and (4) functional coupling of rsEEG alpha rhythms among anterior and posterior cortical areas. These findings suggest that the traditional in-vivo neuroimaging biomarkers of structural and functional brain connectivity may offer an incomplete functional picture of the effects of AD neuropathology/neurodegeneration without a better understanding of the disease implications on those neurophysiological oscillatory mechanisms involving the posterior DMN hubs for the regulation of cortical arousal and quiet vigilance. In line with a broader view of Precision Medicine, combined structural MRI and rsEEG biomarkers in the DMN may represent new promising endpoints for the assessment and intervention of the effects of AD on cortical networks underpinning arousal and quiet vigilance (Teipel et al. 2016).

### The “dark side” of the biomarker panel for the AD assessment

The present results and the above speculative explanation motivate further investments and research aimed at testing the beneficial heuristic and clinical effects of the inclusion of rsEEG measures in the actual panel for the assessment of AD patients, namely the A-T-N(C) Framework (Jack Jr et al. 2018). Those rsEEG measures may be pathophysiological “P” biomarkers in the A-T-N(C) Framework. In an extended A-T-N(C) Framework, the pathophysiological (P) rsEEG biomarkers may be placed between those for the tauopathy (T) and the neurodegeneration (N), as the relative neurophysiological mechanisms generating rsEEG rhythms may be deranged even before the neural loss. With this update, the A-T–P-N Framework may enlighten the “dark side” of the actual instrumental assessment in AD patients (Babiloni 2022), and the dream of Hans Berger (the “father” of human EEG) may come true after almost 100 years ago from his first rsEEG recording.

### Methodological remarks

In interpreting the present results, the pros and cons of the present methodological approach should be considered. Here we used the MNI152 cortical source model implemented in the official eLORETA freeware (https://www.uzh.ch/keyinst/loreta). That model was originally built from high-resolution structural T1-weighted MRIs recorded in 152 adults (86 males and 66 females) aged 18–44 years (Evans et al. 2012) and is commonly used as a head template in neuroimaging and EEG studies. Notably, this study’s participants were 57–87 years old, so some differences in the head volume are expected (Fillmore et al. 2015). At this early stage of the research, we used the MNI152 model over individual MRIs to facilitate the replicability of the results and discussion with reference to several previous field studies (Babiloni et al. 2020, 2021). Furthermore, we did it based on the following additional considerations: (1) high-resolution functional neuroimaging studies in patients typically use a unique MRI-based brain model as a template constructed by averaging the brain structural MRIs of all participating patients and controls; (2) the rsEEG rhythms are intrinsically generated by largely distributed cortical sources; (3) eLORETA is characterized by spatially smoothing rsEEG source estimates (the reason for its name of “low-resolution brain electromagnetic tomography”); and (4) here the eLORETA rsEEG source estimates were averaged within relatively large cortical ROIs, thus further reducing the spatial resolution of the rsEEG source solutions. The general low resolution of the present rsEEG estimation is expected to mitigate the effects of the variability in the individual brains across middle and old ages.

Another methodological limitation of this study concerns the harmonization of the experimental procedures. All clinical units of this study belong to the PDWAVES Consortium and followed local protocols for biofluid sampling, resting-state EEG recordings, structural MRI scans, and clinical and neuropsychological testing in agreement with the procedures recommended by the Consortium “Key” researchers (www.pdwaves.eu). However, we used neither homemade videos/focus group meetings for a fine procedural harmonization across the clinical units nor identical settings for the rsEEG and neuroimaging data collection. Furthermore, there were (minor) differences in the instructions for the participants or in the instrumental recording settings across the clinical units. Due to these sources of variability, we stated that this study cannot be considered a well-controlled sponsored clinical trial. To mitigate the effects of those variability sources, we implemented the following procedures agreed upon by the “Key” researchers of the PDWAVES Consortium (www.pdwaves.eu): (1) the centralization of the analysis of the structural MRI and rsEEG data at the Sapienza University of Rome Unit; (2) quality control and qualification of all individual structural MRI and rsEEG datasets; and (3) use of standard operating procedures for the biomarker extraction double checked by 2 experimenters. In addition, we followed the indication of the Reviewer and used the “recording site” as a covariate in all the statistical analyses to account for that source of variability.

Due to the above methodological limitations, the present results motivate future studies using the following step-forward design and procedures: (1) A prospective, longitudinal design may be used to follow the effects of the disease progression on the present relationship between the rsEEG alpha source activity in the DMN, the structural integrity of the DMN, and cognitive status in ADMCI patients; (2) Fully harmonized procedures for all clinical and instrumental data recordings were expected to cross-validate and extend the above relationship; (3) Higher number of scalp electrodes, digitization of the individual electrode montage, its integration within individual MRI-based cortical source spaces, and principal component analysis of the rsEEG solutions may enhance the spatial resolution of those solutions and unveil further disruptive disease effects on the neurophysiological oscillatory mechanisms generating rsEEG rhythms and regulating vigilance; and (4) Combined estimation of rsEEG source activity/connectivity and resting-state functional MRI may reveal the extent to which the abnormalities in the rsEEG alpha source activities estimated in the posterior DMN hubs may impact the general topology of the functional connectivity in the resting-state cortical neural networks. Furthermore, recording functional MRI during an attention task would allow testing the hypothesis that in ADD patients, the posterior DMN hubs showing poor rsEEG alpha source activity may be characterized by poor BOLD task-related de-activation, unveiling a loss of DMN function.

## Conclusions

In this exploratory study, we tested the hypothesis of a specific relationship between 2 typical AD features: the integrity in the DMN and the “slowing” of the rsEEG rhythms. For this purpose, clinical and instrumental datasets were available in an international archive (www.pdwaves.eu). The main results showed a significant positive association between the GM volume in the DMN, the rsEEG alpha source activity estimated in the posterior DMN nodes (parietal and PCC), and the global cognitive status (MMSE score) in the Nold and ADD participants. Compared with the Nold group, the ADD group showed lower DMN GM, lower rsEEG alpha source activity in those nodes, and lower global cognitive status. These results suggest that the DMN structural integrity and the rsEEG alpha source activities estimated in the DMN posterior hubs might be related and predict the global cognitive status in ADD and Nold persons.

## Supplementary Material

Supplementary_Materials_bhad300

## Data Availability

Datasets are available under scientific agreement with the corresponding author.
