# Supplementary material for: Relationship between default mode network and resting-state electroencephalographic alpha rhythms in cognitively unimpaired seniors and patients with dementia due to Alzheimer’s disease
Source: Cereb Cortex. 2023 Aug 23;33(20):10514–27. doi: 10.1093/cercor/bhad300 (PMC10588004; doi:10.1093/cercor/bhad300)
Supplement: Supplementary_Materials_bhad300 [file supplementary_materials_bhad300.docx]

**Supplementary Materials**

**RELATIONSHIP BETWEEN DEFAULT MODE NETWORK AND RESTING-STATE ELECTROENCEPHALOGRAPHIC ALPHA RHYTHMS IN COGNITIVELY UNIMPAIRED SENIORS AND PATIENTS WITH DEMENTIA DUE TO ALZHEIMER'S DISEASE**

Claudio Babiloni^1,2*¥^, Susanna Lopez^1¥^, Giuseppe Noce^3^, Raffaele Ferri^4^, Simonetta Panerai^4^, Valentina Catania^4^, Andrea Soricelli^3,5^, Marco Salvatore^3^, Flavio Nobili^6,7^, Dario Arnaldi^6,7^, Francesco Famà^6^, Federico Massa^6^, Carla Buttinelli^8^, Franco Giubilei^8^, Fabrizio Stocchi^9^, Laura Vacca^9^, Moira Marizzoni^10^, Fabrizia D'Antonio^11^, Giuseppe Bruno^11^, Carlo De Lena^11^, Bahar Güntekin^12^, Ebru Yıldırım^13^, Lutfu Hanoğlu^14^, Görsev Yener^15^, Deniz Yerlikaya^16^, John Paul Taylor^17^, Julia Schumacher^17,18^, Ian McKeith^17^, Laura Bonanni^19^, Patrizia Pantano^11,20^, Claudia Piervincenzi^11^, Nikolaos Petsas^21^, Giovanni B. Frisoni^10,22^, Claudio Del Percio^1^ ,

and Filippo Carducci^1^

*^1^ Department of Physiology and Pharmacology "Vittorio Erspamer,” Sapienza University of Rome, Rome, Italy;*

*^2^ Hospital San Raffaele Cassino, Cassino (FR), Italy;*

*^3^* *IRCCS Synlab SDN, Naples, Italy;*

*^4^ Oasi Research Institute - IRCCS, Troina, Italy;*

*^5^ Department of Motor Sciences and Healthiness, University of Naples Parthenope, Naples, Italy;*

*^6^ Clinica neurologica, IRCCS Ospedale Policlinico San Martino, Genova, Italy;*

*^7^ Dipartimento di Neuroscienze, Oftalmologia, Genetica, Riabilitazione e Scienze Materno-infantili (DiNOGMI), Università di Genova, Italy;*

*^8^ Department of Neuroscience, Mental Health and Sensory Organs, Sapienza University of Rome, Rome, Italy;*

^9^ *IRCCS San Raffaele, Rome, Italy;*

*^10^ Laboratory of Alzheimer's Neuroimaging and Epidemiology, IRCCS Istituto Centro San Giovanni di Dio Fatebenefratelli, Brescia, Italy;*

*^11^ Department of Human Neurosciences, Sapienza University of Rome, Rome, Italy;*

*^12^ Department of Biophysics, International School of Medicine, Istanbul Medipol University, Istanbul, Turkey;*

*^13^ Program of Electroneurophysiology, Vocational School, Istanbul Medipol University, Istanbul, Turkey;*

*^14^ Department of Neurology, School of Medicine, Istanbul Medipol University, Istanbul, Turkey;*

*^15^ Izmir School of Economics, Faculty of Medicine, Izmir, Turkey;*

*^16^ Health Sciences Institute, Department of Neurosciences, Dokuz Eylül University, Izmir, Turkey;*

*^17^ Translational and Clinical Research Institute, Faculty of Medical Sciences, Newcastle University, UK;*

*^18^ German Center for Neurodegenerative Diseases (DZNE), Rostock, Germany;*

*^19^ Department of Medicine and Aging Sciences, University “G. d'Annunzio” of Chieti-Pescara, Chieti, Italy;*

*^20^ IRCCS Neuromed, Pozzilli (IS), Italy;*

*^21^ Scuola di Specializzazione in Statistica Medica e Biometria, Dipartimento di Sanità Pubblica e Malattie Infettive, Sapienza University of Rome, Rome, Italy;*

*^22^ Memory Clinic and LANVIE - Laboratory of Neuroimaging of Aging, University Hospitals and University of Geneva, Geneva, Switzerland.*

**Supplementary Materials and Methods**

Details about the healthy cognitively unimpaired (Nold) and patients with dementia due to Alzheimer’s disease (ADD) participant cohort (“*Participants*“) and to the procedure of the rsEEG (“*The rsEEG recordings”, “Preliminary rsEEG data analysis”, Spectral analysis of the rsEEG epochs”)* and MRI data acquisition and preprocessing (*“Magnetic Resonance Imaging (MRI) data acquisition and anatomical preprocessing”, “Cortical network parcellation”)* are reported in the corresponding sections of the main manuscript.

*Computation of the resting-state eyes-closed electroencephalographic (rsEEG) posterior source activities by eLORETA*

As a control analysis, we collapsed the eLORETA solutions as calculated in the main manuscript “*Materials and Methods*” (section *“Cortical sources of rsEEG rhythms in the DMN, SMN, and DAN as computed by eLORETA”),* in six cortical regions of interest (ROIs). The correspondence between the BAs and the ROIs for the present study is reported in Table Supplementary Material 1 (SM1).

| **BRODMANN AREAS** **INTO THE REGIONS OF INTEREST (ROIs)** | |
| --- | --- |
| **Frontal** | 8, 9, 10, 11, 44, 45, 46, 47 |
| **Central** | 1, 2, 3, 4, 6 |
| **Parietal** | 5, 7, 30, 39, 40, 43 |
| **Occipital** | 17, 18, 19 |
| **Temporal** | 20, 21, 22, 37, 38, 41, 42 |
| **Limbic** | 31, 32, 33, 34, 35, 36 |

***Table Supplementary Material 1 (SM1).*** *Regions of interest (ROIs) used for the estimation of the cortical sources of the resting state eyes-closed electroencephalographic (rsEEG) rhythms in the present study. Each ROI is defined by some Brodmann areas of the cerebral source space in the freeware used in this study, namely the exact low-resolution brain electromagnetic source tomography (eLORETA).*

The normalization procedure and the frequency resolution of 0.5 Hz were the same as the main analysis.

In line with the general low spatial resolution of the current EEG methodological approach (i.e., 30 scalp electrodes), we performed a regional analysis of the eLORETA solutions. For this purpose, we collapsed the eLORETA solutions within frontal, central, parietal, occipital, temporal, and limbic macro-regions (ROIs) considered separately. Table 2 reports the list of the BAs used for the ROIs evaluated in the present study. Of note, the main advantage of the regional analysis of eLORETA solutions was that we could disentangle the rsEEG source activity in contiguous cortical areas. For example, the rsEEG source activity in the occipital ROI was separated from that estimated in the parietal and temporal ROIs, etc. This was possible because eLORETA solves the linear inverse problem by considering (at least in part) the effects of the head as a volume conductor. In contrast, the solutions of rsEEG power density computed at a parietal scalp electrode reflect the contribution of source activities not only of the underlying parietal cortex but also of surrounding occipital and temporal cortices.

The statistical session tested the hypothesis that the posterior rsEEG source activities may differ between the Nold and ADD groups. To this aim, an ANOVA was computed using the average among the parietal, temporal, and occipital normalized eLORETA solutions as a dependent variable. The ANOVA factors were Group (Nold, ADD) and Band (delta, theta, alpha 2, alpha 3, mean between beta 1 and beta 2, beta 1-2). The confirmation of this control hypothesis may require: (1) a statistically significant ANOVA interaction including the factor Group (p < 0.05) and (2) a post-hoc Duncan test indicating statistically significant (p < 0.05, Bonferroni corrected) differences in the posterior rsEEG source activities between the Nold and ADD groups at the delta and alpha bands.

*Correlation analysis between the MRI markers and posterior rsEEG source activity*

We evaluated the control hypothesis that the MRI markers of the DMN, SMN, and DAN were related to the rsEEG markers in Nold and ADD participants, considered as a whole group. To address this aim, we performed a correlation analysis by the Pearson test (p < 0.05, Bonferroni corrected) between the normalized gray matter, GM, volume (i.e., normalized DMN, SMN, and DAN volumes considered separately) and the rsEEG posterior source activities (i.e., average among parietal, occipital, and temporal delta, theta, alpha 2, alpha 3, beta 1-2 eLORETA solutions) in the Nold and ADD participants as a whole group.

*Association between neurodegeneration (MRI markers), global cognition (mini mental state evaluation, MMSE, score), and pathophysiology (rsEEG posterior source activities)*

We evaluated the association between the MRI marker of the DMN, SMN and DAN (normalized GM volume), the rsEEG posterior source activities showing statistically significant differences (p < 0.05) between Nold and ADD groups, and the global cognition (mini mental state evaluation, MMSE, score) with several correlation analysis (Pearson test, p < 0.05) in the Nold and ADD participants as a whole group.

**Supplementary Results**

*Distribution of the rsEEG posterior source activities in Nold and ADD groups*

The results the rsEEG source activities in all Nold and ADD participants are illustrated in Figure SM1. This Figure shows the mean values (± SE, Log10 transformed) of the posterior rsEEG (i.e., average among the parietal, temporal, and occipital normalized eLORETA solutions) source activities between the Nold (N = 40) and ADD (N = 45) groups. The distribution of those rsEEG source activities differed across the Groups and the Bands. In the Nold group, as a physiological reference, the temporal, parietal, and occipital (eLORETA) alpha 2 and 3 source activities showed dominant values over the other frequency bands. Delta and theta posterior rsEEG source activities were characterized by relatively low values, while the beta1-2 (mean between the beta 1 and the beta 2) source activities were generally very low. Compared to the Nold group, the ADD group exhibited a substantial decrease in the posterior alpha 2 and alpha 3 rsEEG source activities. Furthermore, the ADD group exhibited an increase in the posterior delta and theta rsEEG source activities. Based on these input data, the ANOVA results showed a statistical interaction effect (F (4, 332) = 11.27, p < 0.0001) among the factors Group (Nold and ADD) and Band (delta, theta, alpha 2, alpha 3, beta 1-2). The Duncan planned post-hoc (p < 0.05 Bonferroni correction for 5 Bands, p < 0.05/5 = 0.01) testing produced the following core results: (1) the discriminant pattern Nold < ADD was fitted by the posterior delta and theta rsEEG source activities (p < 0.002 and p < 0.01, respectively); and (2) the discriminant pattern Nold > ADD was fitted by the posterior alpha 2 and alpha 3 rsEEG source activities (p < 0.01). Of note, these findings were not due to outliers from those individual regional normalized eLORETA current densities (log 10 transformed), as shown by the results of the Grubbs’ test set with an arbitrary threshold of p > 0.001.

**
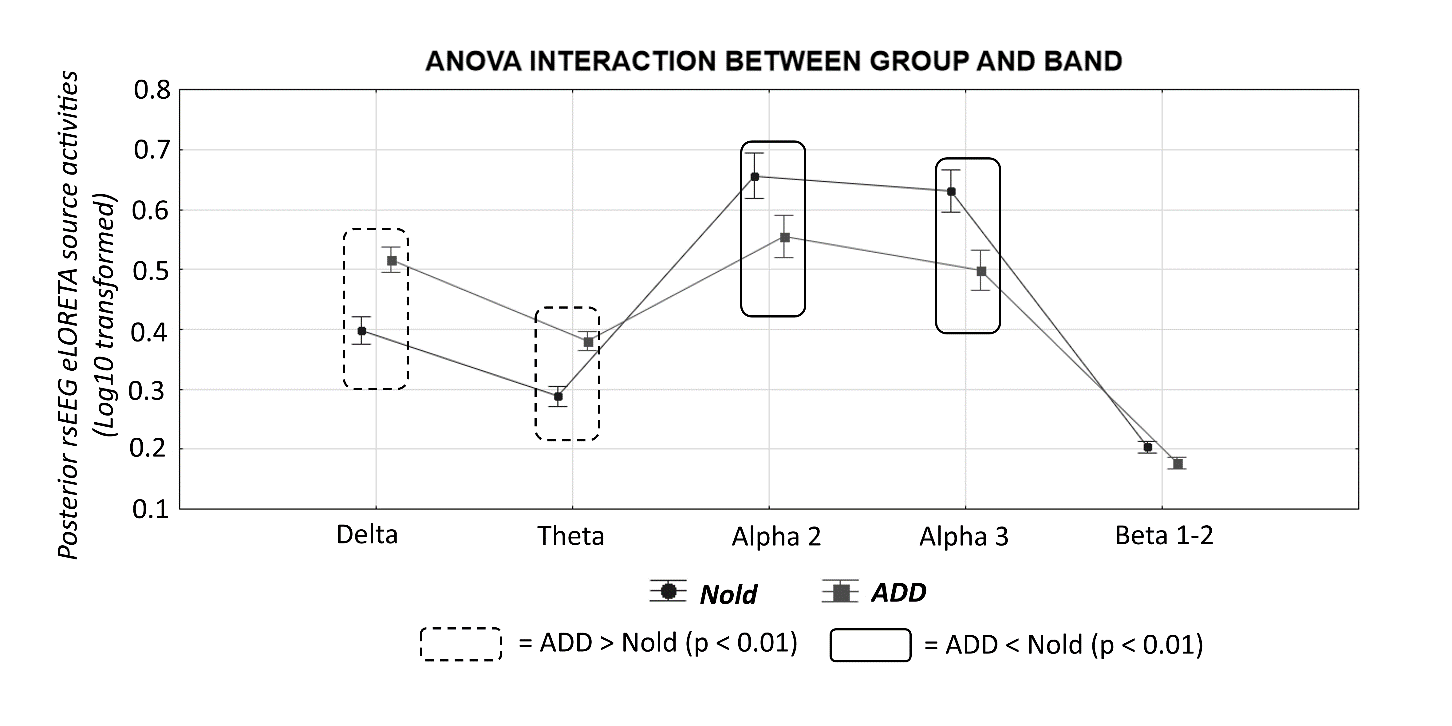
*Figure Supplementary Material 1 (SM1).*** *Posterior normalized eLORETA solutions (mean across subjects ± standard error, SE, log 10 transformed) of the resting-state eyes-closed electroencephalographic (rsEEG) source activities relative to a statistical ANOVA interaction among the factors Group (Nold, N = 40; ADD, N = 45) and Band (delta, theta, alpha2, alpha3, beta 1-2). This ANOVA design used the average among the parietal, occipital, and temporal rsEEG eyes-closed normalized eLORETA solutions as a dependent variable. The rectangles indicate the cortical regions and frequency bands in which the posterior eLORETA solutions (rsEEG source activities) presented a statistically significant pattern Nold ≠ ADD (p < 0.05 Bonferroni corrected).*

*Correlation analysis between the MRI markers and posterior rsEEG source activity*

Pearson’s test (p < 0.05 Bonferroni correction for 3 Networks X 5 rsEEG Bands, p < 0.05/15 =0.0033; see Figure SM2 and Table SM2) evaluating the relationship between the MRI and rsEEG markers showed the following statistically significant negative correlations (p < 0.05 corrected): (i) the normalized DMN volume vs the posterior (average among parietal, occipital, and temporal) delta rsEEG source activity (r = -0.296, p < 0.003); and (ii) that DMN volume vs the posterior (average among parietal, occipital, and temporal) theta rsEEG source activity (r = -0.33, p < 0.001). The higher the DMN volume, the lower the delta and theta eLORETA source activities. No other correlation reached the statistical threshold.

**
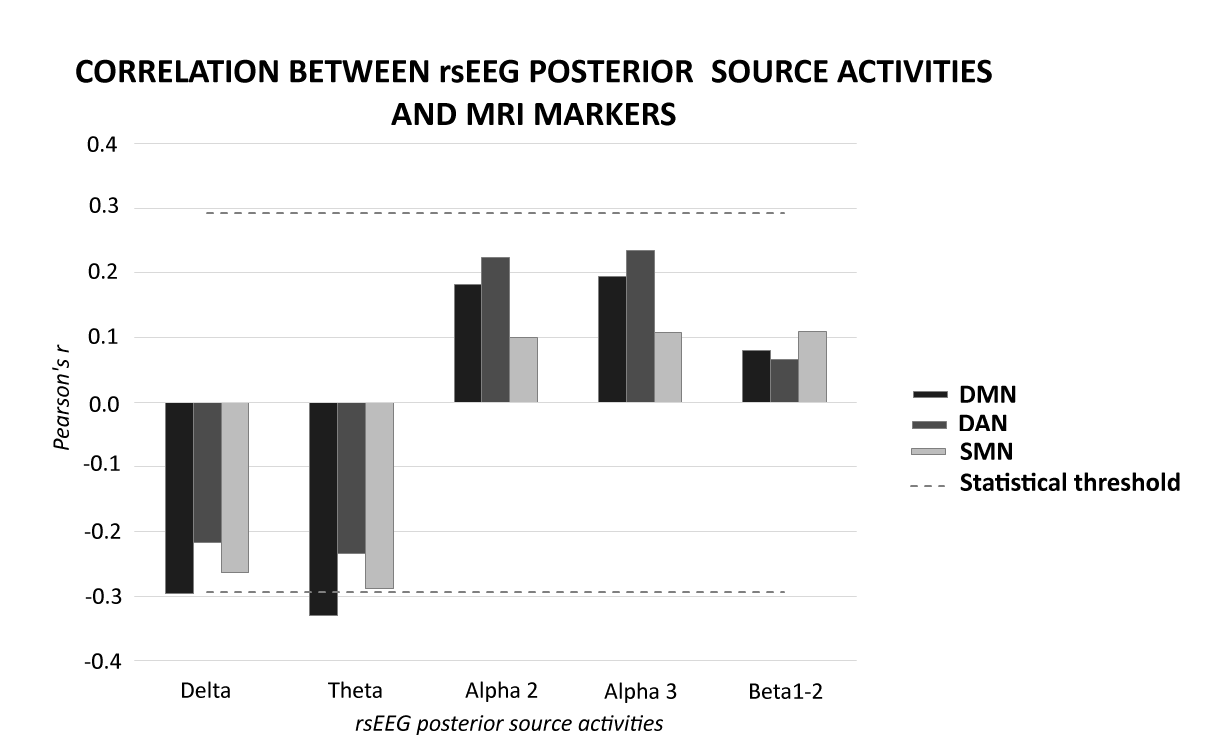
**

***Figure SM2.*** *Pearson’s r values quantifying the correlation between posterior (i.e., average among parietal, occipital, and temporal) rsEEG source activities and MRI markers (GM volume of DMN, DAN, and SMN). The statistical threshold equivalent to Bonferroni correction for 3 Networks X 5 rsEEG Bands (p < 0.05/15 =0.0033) is illustrated.*

| **Cortical Network** | **Delta OTP** | **Theta OTP** | **Alpha2 OTP** | **Alpha3 OTP** | **Beta1-2 OTP** |
| --- | --- | --- | --- | --- | --- |
| **DMN** | **r = -0.296**  **p = 0.003** | **r = -0.33**  **p = 0.001** | r = 0.182  p = 0.047 | r = 0.195  p = 0.037 | r = 0.080  p = 0.233 |
| **DAN** | r = -0.217  p = 0.023 | r = -0.234  p = 0.015 | r = 0.223  p = 0.020 | r = 0.234  p = 0.016 | r = 0.066  p = 0.273 |
| **SMN** | **r = -0.264**  **p = 0.007** | **r = -0.289**  **p = 0.004** | r = 0.100  p = 0.180 | r = 0.108  p = 0.162 | r = 0.109  p = 0.161 |

***Table SM2.*** *Results of the correlation analysis (Pearson’s r and p-values) between MRI markers and posterior rsEEG source activities. Statistically significant correlations are in bold according to the Bonferroni correction (p < 0.05 Bonferroni correction for 3 Networks X 5 rsEEG Bands, p < 0.05/15 =0.0033).*

*Association between neurodegeneration (magnetic resonance imaging, MRI, markers), global cognition (mini mental state evaluation, MMSE, score), and pathophysiology (delta and theta posterior resting-state eyes-closed electroencephalographic, rsEEG, source activities)*

Figure SM3 illustrates the scatterplots revealing the association between the markers of the following relevant variables measured in healthy cognitively unimpaired (Nold) persons and patients with dementia due to Alzheimer’s disease (ADD) considered as a whole group:

- Global cognition (mini mental state evaluation, MMSE, score) and neurodegeneration in the cortical default mode network, DMN, as revealed by structural magnetic resonance imaging, MRI (normalized DMN gray matter, GM, volume; left);
- Delta resting-state eyes-closed electroencephalographic, rsEEG, posterior source activities and neurodegeneration in the DMN (upper middle);
- Delta rsEEG posterior source activities and global cognition (MMSE; upper right);
- Theta rsEEG posterior source activities and neurodegeneration in the DMN (lower middle);
- Theta rsEEG posterior source activities and global cognition (MMSE; lower right).

**
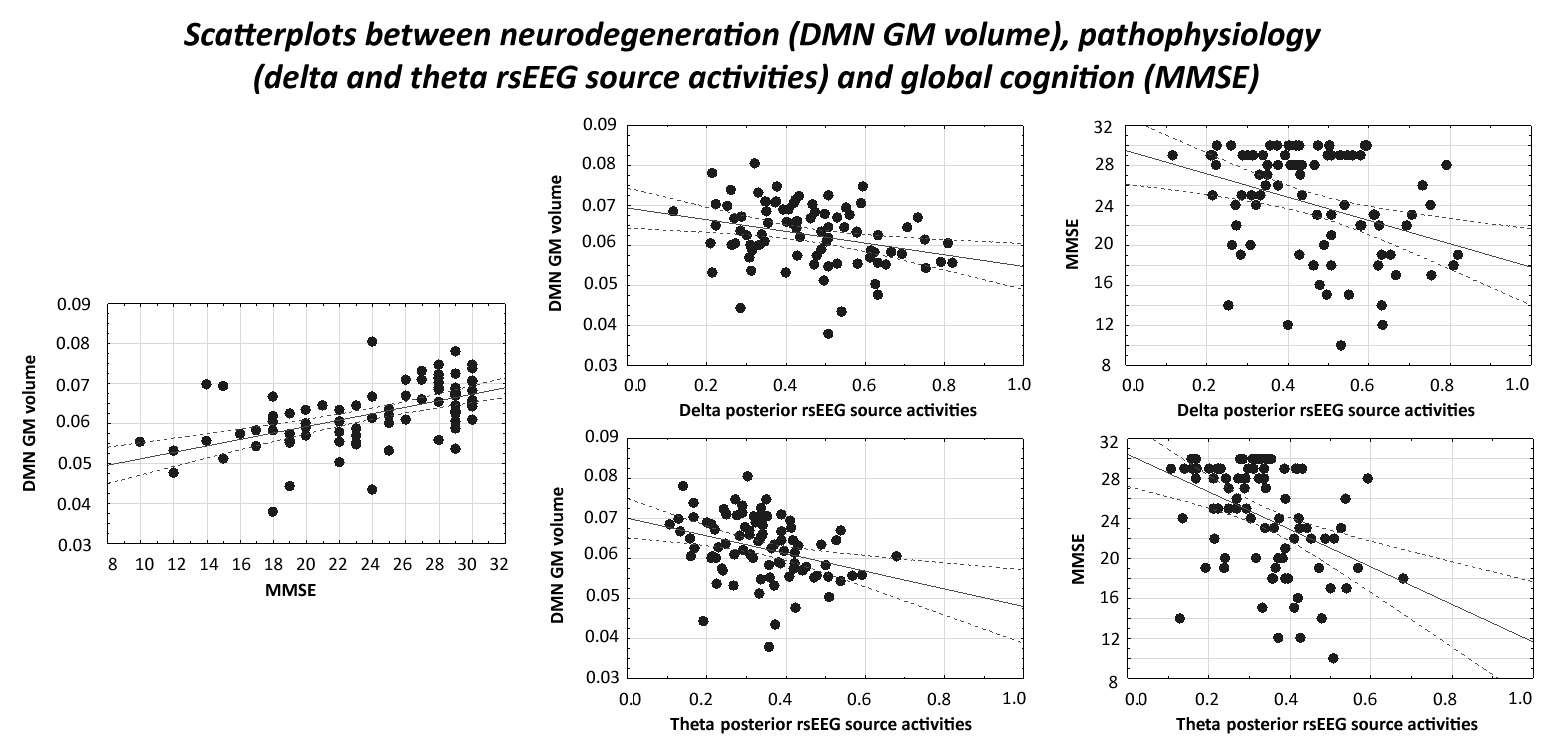
**

***Figure SM3.*** *Scatterplots illustrating the association between the following variables measured in healthy cognitively unimpaired (Nold) persons and patients with dementia due to Alzheimer’s disease (ADD): global cognition (Mini Mental State Evaluation, MMSE, score) and neurodegeneration in the cortical default mode network (DMN; normalized DMN gray matter, GM, volume; left); resting-state eyes-closed electroencephalographic (rsEEG) posterior delta source activities and neurodegeneration in the DMN (upper middle); rsEEG posterior delta source activities and global cognition (MMSE; upper right); rsEEG posterior theta source activities and neurodegeneration in the DMN (lower middle); rsEEG posterior theta source activities and global cognition (MMSE; lower right). In this analysis, the Nold and ADD subject were considered as a whole group.*

The Figures SM4 and SM5 illustrate the scatterplots revealing the association between the markers of the global cognition (MMSE), rsEEG delta and theta source activities, and the cortical dorsal attention network (DAN) and sensorimotor network (SMN), as revealed by structural MRI.

**
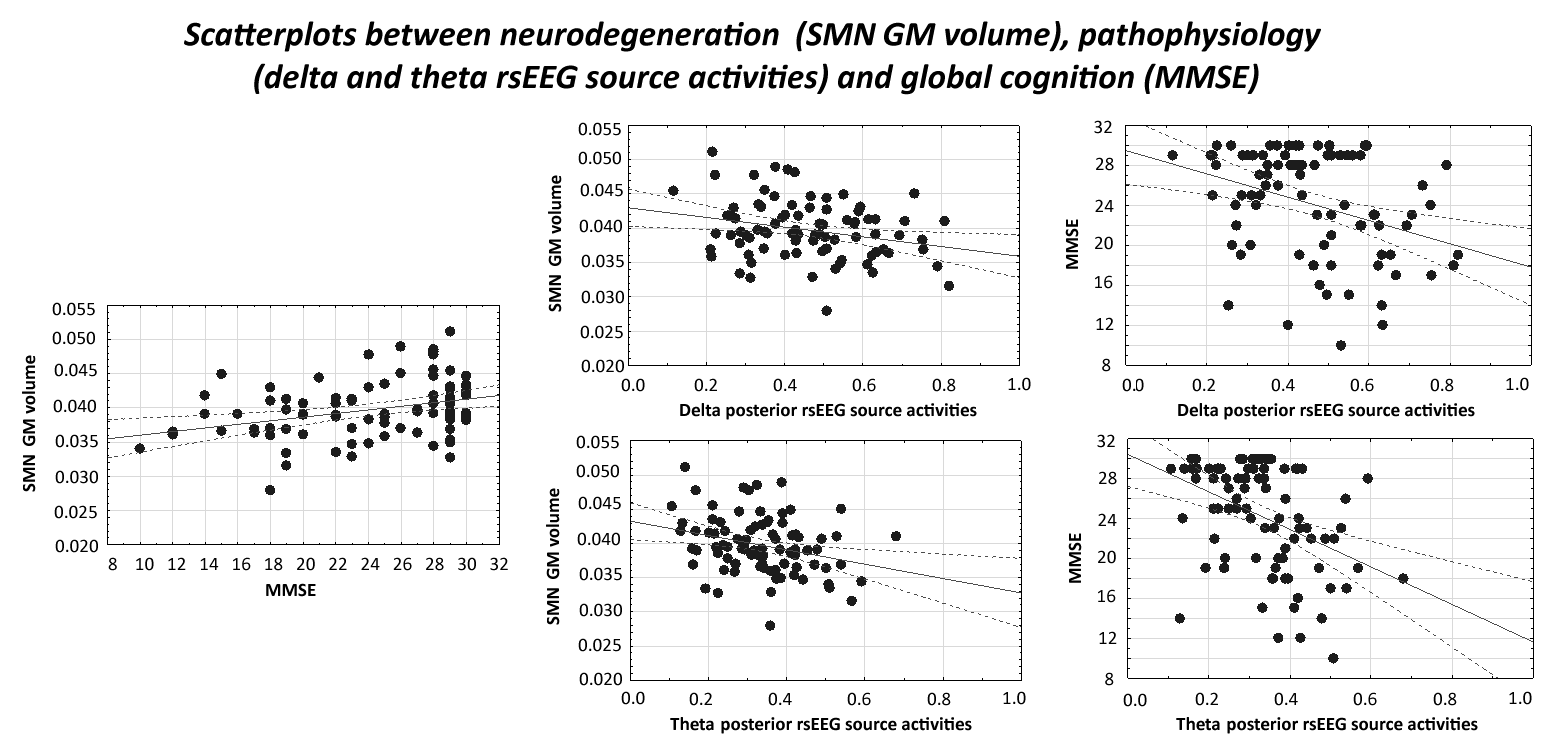
**

***Figure SM4.*** *Scatterplots illustrating the association between the following variables measured in the Nold persons and ADD patients: global cognition (MMSE) and neurodegeneration in cortical dorsal attention network (DAN; normalized DAN GM volume; left); rsEEG posterior delta source activities and neurodegeneration in the DAN (upper middle); rsEEG posterior delta source activities and global cognition (MMSE; upper right); rsEEG posterior theta source activities and neurodegeneration in the DAN (lower middle); rsEEG posterior theta source activities and global cognition (MMSE; lower right). In this analysis, the Nold and ADD subject were considered as a whole group.*

*
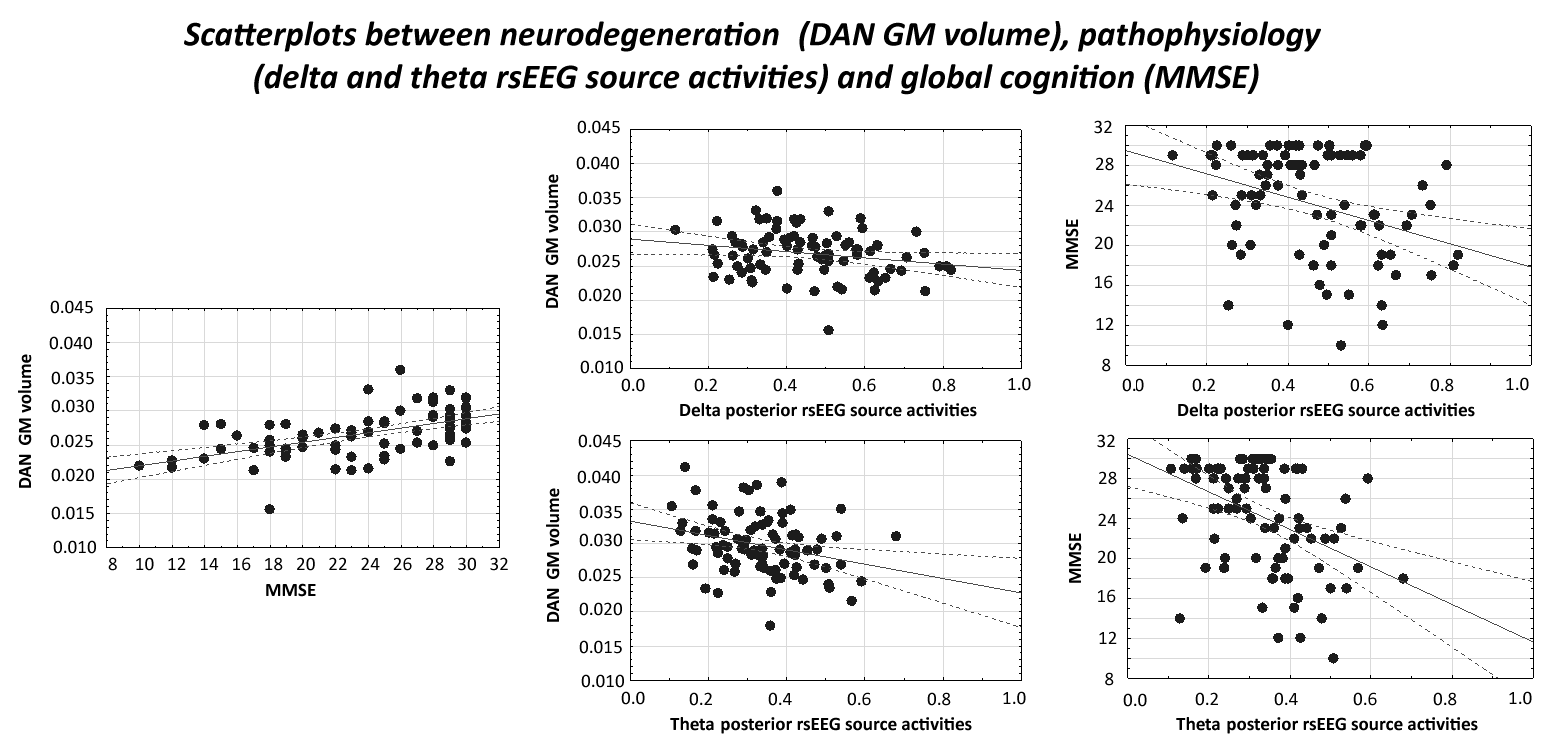
*

***Figure SM5.*** *Scatterplots illustrating the association between the following variables measured in the Nold persons and ADD patients: global cognition (MMSE) and neurodegeneration in the cortical sensorimotor network (SMN; normalized SMN GM volume; left); rsEEG posterior delta source activities and neurodegeneration in the SMN (upper middle); rsEEG posterior delta source activities and global cognition (MMSE; upper right); rsEEG posterior theta source activities and neurodegeneration in the SMN (lower middle); rsEEG posterior theta source activities and global cognition (MMSE; lower right). In this analysis, the Nold and ADD subject were considered as a whole group.*

Table SM3 reports the Pearson’s r (for the above MRI markers and the rsEEG delta and theta source activities) and the Spearman R (for analysis involving the MMSE score), as well as the p-values for the correlation between pairs of the mentioned variables.

|  | **Delta rsEEG source activity** | **Theta rsEEG source activity** | **MMSE** |
| --- | --- | --- | --- |
| **DMN GM volume** | r = -0.2964  p = 0.006 | r = -0.3304  p = 0.002 | R = 0.5767  p = 0.00001 |
| **SMN GM volume** | r = -0.2964  p = 0.006 | r = -0.3304  p = 0.002 | R = 0.3141  p = 0.003 |
| **DAN GM volume** | r = -0.2964  p = 0.006 | r = -0.3304  p = 0.002 | R = 0.5656  p = 0.00001 |
| **MMSE** | R = -0.3505  p = 0.001 | R = -0.4408  p = 0.00002 | - |

***Table SM3.*** *Results of the correlation analysis as revealed by Pearson’s r between the following variables measured in healthy cognitively unimpaired (Nold) persons and patients with dementia due to Alzheimer’s disease (ADD): (1) normalized gray matter (GM) volume in the cortical default mode (DMN), dorsal attention (DAN), and sensorimotor (SMN) networks as revealed by structural magnetic resonance imaging; (2) resting-state eyes-closed electroencephalographic (rsEEG) posterior source activities at delta (< 4 Hz) and theta (about 4-7 Hz); and (3) global cognitive status as revealed by Mini Mental State Evaluation (MMSE) score. The statistically significant p-values (p < 0.05) are reported for each pair of variables.*
